# Dietary Ecology of Murinae (Muridae, Rodentia): A Geometric Morphometric Approach

**DOI:** 10.1371/journal.pone.0079080

**Published:** 2013-11-13

**Authors:** Ana Rosa Gómez Cano, Manuel Hernández Fernández, M. Ángeles Álvarez-Sierra

**Affiliations:** 1 Departamento de Paleontología, Facultad de Ciencias Geológicas, Universidad Complutense de Madrid, Madrid, Spain; 2 Departamento de Geología Sedimentaria y Cambio Medioambiental, Instituto de Geociencias (UCM, CSIC), Madrid, Spain; Team ‘Evo-Devo of Vertebrate Dentition’, France

## Abstract

Murine rodents represent a highly diverse group, which displays great ecological versatility. In the present paper we analyse the relationship between dental morphology, on one hand, using geometric morphometrics based upon the outline of first upper molar and the dietary preference of extant murine genera, on the other. This ecomorphological study of extant murine rodents demonstrates that dietary groups can be distinguished with the use of a quantitative geometric morphometric approach based on first upper molar outline. A discriminant analysis of the geometric morphometric variables of the first upper molars enables us to infer the dietary preferences of extinct murine genera from the Iberian Peninsula. Most of the extinct genera were omnivore; only *Stephanomys* showed a pattern of dental morphology alike that of the herbivore genera.

## Introduction

Rodentia is the most speciose group of mammals and the morphology of their dentition is highly morphologically specialized [1]. Addressing different rodent groups in detail, one can observe that the diversity of each group can also be seen in the disparity of their morphological dental features [2]. Furthermore, the morphological characters could be directly related with the ecological features of each taxon such as its habitat or diet [3]–[5].

Several authors had demonstrated an interesting relationship between dental features and grazing diet in rodents, inferred by classic morphometric methodologies in lateral view (hypsodonthy) [6] or in occlusal pattern [7]. Other studies have pointed out the existence of a close relationship between feeding habits and skulls or mandibles morphology using classical and geometric morphometric tools for quantification of shapes [8]–[12]. To work with fossil material, however, there is a need to establish a methodology based on the study of isolated teeth because these pieces are the most abundant in the fossil sites where rodents are recorded, particularly in the case of the fossil record of the European Miocene. The geometric morphometric methodologies are of great interest because they enable researchers to quantify the variation in shape and size to develop morphospace, which facilitate the ecological and evolutionary inferences [13], [8]–[10], [14].

Previous research on geometric morphometrics of rodent molars provides interesting results with regard to describing ecological preferences [7], [15], [9], [16]. The interest of our study is based upon the use of a methodological approach allowing us to analyse the high morphological diversity within extant and extinct murine rodents and to associate it with their feeding habits. Murines are the largest subfamily of muroid rodents [17]. Furthermore, the wide range of habitats and the large amount of studies on extant and fossil taxa [18], [7], [19], [20], [17], [2] of this group makes it an interesting one for our study.

## Materials and Methods

To developed the study one of the authors (ARGC) visited the Murinae collections at the Museum National d'Histoire Naturelle in Paris (France) during August 2010, to take photos of the first upper molars by the authorization of the Christiane Denys curator of the Rodentia section of Mammalian Department and supervised by Dr. Emmanuelle Stoetzel.

### Dietary Categories

Based on descriptions available from the literature (Appendix S1 and references therein) we classified each extant genus into one of three dietary categories [21], [10]: 1) herbivores (n = 22) if it feeds mostly on plant matter being nearly purely herbivorous; 2) omnivores (n = 40), when it includes both animal-dominated and plant-dominated taxa; or 3) and faunivores (n = 14), with a diet composed primarily of animal matter, being nearly purely faunivorous (Appendix S1).

Although these dietary categories are a simplification of a complex classification of diets, this categorization has been shown to be useful for examining the relationships between morphology and feeding habits [10].

### Samples

When ecomorphological studies focus on supraspecific taxa (genera, families, etc.), they can help to reveal the development of diversity in different groups and understand the course of their adaptive evolution [22].

In this study we included material from extant murines from all over the world and extinct Iberoccitanian murine rodents (Appendix S2 and references therein). We chose the first upper molar (M1) based on its highly distinctive features and it is very useful in studies of fossil material [7].

The samples of extant material included in this paper are housed in the Museum National d’Histoire Naturelle (MNHN), Paris. We photographed the first upper molars of all the murine genera available at the MNHN using a Nikon D300s camera fitted with a Nikon AF-S VR 105 mm f/2.8 IF-ED lens. Furthermore, for those extant genera unavailable in the MNHN collections we included scaled photographs from the literature. In general terms, we compiled a database of 232 specimens of right or left first upper molars of extant Murinae (Appendix S2).

Finally, based on both the information on diets available in the bibliography and the specimens available in the collections and literature, we were able to include in our study 76 of the 124 extant genera of Murinae (61.3%) according to the taxonomic revision of [17].

Moreover, in our analysis we included pictures of teeth belonging to the 9 extinct genera of murine (*Anthracomys*, *Castillomys*, *Castromys*, *Huerzelerimys*, *Occitanomys*, *Paraethomys*, *Progonomys*, *Rhagapodemus* and *Stephanomys*; Appendix S2 and references therein), which have been described at the Iberoccitanian (south western Europe) fossil sites across the whole Upper Miocene [23]. As for the extant taxa, we developed another photographic database for the fossil genera. In this case we compiled pictures of first upper molars from the literature. The intensive sampling work at the Iberoccitanian fossil sites and the large amount of detailed studies of these materials over the last decade [24], enabled us to include 169 scaled pictures and drawings, which represent all the fossil genera and most of the species described in our study area (see Appendix S2 for specimen information and references).

### Morphological Analysis of the Outline

We chose the outline analysis to describe the morphology of the molars because, besides being effective with regard to describing the location of the tubercles characteristic of the murines molar, it is less sensitive to modifications of the dental pattern occurring with wearing than the landmark analysis [13], [25]. Furthermore, whereas individual homologous landmarks are difficult to pinpoint from one molar to another, outline methods have been suggested as be useful tools for the analysis of biological shapes in the absence of homologous landmarks [26].

The molar outline is defined as the two-dimensional projection of the molar viewed from its occlusal side [25]. Following [13] these outlines were digitalized for each tooth as x and y coordinates of sixty-four points equally spaced along the tooth outline with TPSdig2 software version 2.16 [27]. The starting point of each outline was defined at the maximum of curvature in the forepart of the tooth. In order to provide a convenient way to get measurements using right and left molars, left molars were reflected in a mirror and measured as right molars [25].

**Figure 1:**
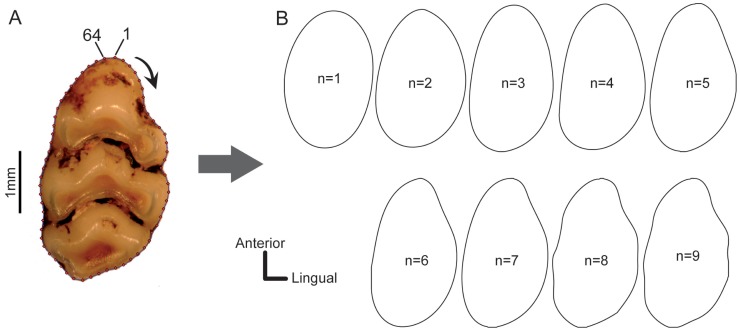
Outline and EFA reconstruction for an increasing number of harmonics. *A*, Outline digitalized from an image of one first upper molar of *Rattus andamanensis* (MNHN 1995 2833) based on 64 points equally distributed using TPSdig2 software version 2.16 [27]; the black arrow indicates the direction of the digitalization process. *B*, Reconstructed outlines for an increasing number of harmonics; n represents the number of harmonics in each outline.

In order to analyse these *x* and *y* coordinates we applied an Elliptic Fourier Analysis (EFA) [28] to the samples data using EFAwin software [29] which extracts Fourier coefficients from the original outline and normalizes these shape variables (Fig. 1). With this method the complex outline can be described as a sum of trigonometric functions, known as harmonics, of decreasing wavelength. Each harmonic is defined by four Fourier Coefficients (FCs), two for the *x* coordinate (A and B) and two for the *y* coordinate (C and D) [30].

Based in our data (Appendix S3) and in previous studies, which demonstrated that the effect of measurement error for upper molars was limited by considering only Fourier Coefficients up to the ninth harmonic [25], [14]. Furthermore, the first harmonic is proportional to the size of each specimen but its four coefficients (A1-D1) are constant due to the standardization [13], [31]. Thus, following previous studies we retain nine harmonics, which represents the best compromise between measurement error and information content [25], [14]. Thus, we finally we retain 36 FCs from these nine harmonics (i.e. A1–D9), which describes the outline of each specimen.

Since our analysis was performed at the genus level, each set of Fourier coefficients describing the outline for each specimen was averaged per genus (Appendix S4).

Finally, as a support for visual interpretation of shape changes we obtained accurate reconstructions of these average outlines using an inverse Fourier transformation [28], [32], which directly provides the Euclidean *xy*-coordinates of the reconstructed outline [33].

### Statistical Analysis

In order to evaluate the importance of among-group differentiation relative to within-group variation using the dietary categories as grouping variables we performed a non-parametric multivariate analysis on variance (NPMANOVA, [34]) on the obtained sets of Fourier Coefficients (A1 to D9). Likewise, we evaluated pairwise NPMANOVAs between all pairs of dietary groups by means of a *post-hoc* test (Bonferroni).

Moreover, associated with the analysis of variance, we estimated canonical functions (Canonical Variate Analysis, CVA [35]), which enabled us to plot scores for each dietary group and to visualize the pattern of morphological differentiation in each of them. The CVA produces maximal and second to maximal separation between all groups, and its axes are linear combinations of the original variables. Statistical analyses were performed with PAST v. 2.17 [36].

Additionally, [37] have linked dietary categories with molar size in a group of Primates. We were able to explore this relationship in murine rodents because the EFA calculates the size variable (measured as major axis length) linked to the first FC. Therefore we analysed the differences in size (M1 length) and shape (allometry) by a linear correlation between size and CV1 and CV2 as shape estimators [38]. Furthermore we analysed the differences in size among the feeding habits through ANOVA and *Post-hoc* Tukey tests using SPSS v. 15.

Finally, for the purpose of classifying the murine extinct genera in the dietary categories we assessed a multivariate discriminant analysis on morphological variables (FCs of their M1 and size) employing SPSS v 15.

## Results and Discussion

### Relation between Shape and Diet

Rather than the high apparently homogeneity of the dentition pattern described for this group [39], we found that shapes defined by the Fourier Coefficients (FC) showed significant differences among the three dietary categories. The FC showed significant differences between dietary categories in the NPMANOVA analysis (F = 6.118; p = 0.0004). The results of the pairwise comparison of Euclidean distance were significant (p<0.05) for all dietary pairs (table 1).

**Table 1:** Pairwise comparison of Euclidean distance.

	Herbivore	Omnivore	Faunivore
Herbivore		0.027	0.001
Omnivore	0.027		0.003
Faunivore	0.001	0.003	

P values obtained in the pairwise comparison, p<0.05 indicates significant differences among the dietary groups.

**Figure 2:**
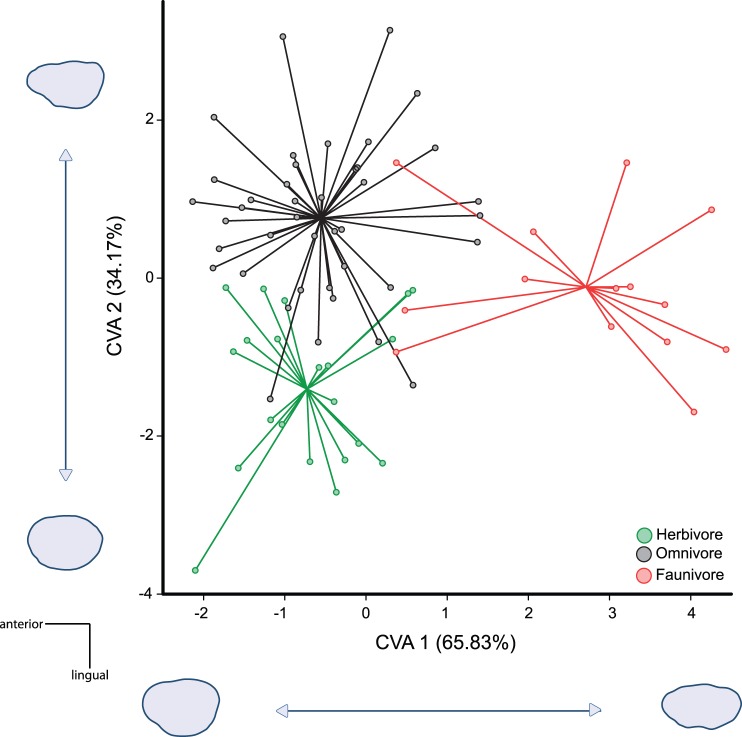
Inter-dietary shape differentiation of the first upper molars. Variation in shapes variation was estimated by the first two axes of a canonical analysis of the EFT Fourier coefficients of the M1 outline. Colours correspond to the different dietary groups. White for herbivores; Grey for omnivores; Black for faunivores. On each axis, shape changes corresponding to the canonical axes are depicted, corresponding to the maximum values of the axes on the plot.

The two axes of the CVA (Fig. 2) explained 65.8% (CV1) and 34.2% (CV2) respectively of total variation in shape of molar outline. Reconstruction of mean outlines corresponding to theoretical outlines equivalent to the coordinates along the canonical axes showed the differences involved in this differentiation of dietary shape.

The first canonical axis (CV1) showed a morphological gradient in the shape of the outline from tight and elliptical molars, with a prominent cusp (t1) in the anterior edge on the positive side of this axis to wide molars on the negative side of this axis (Fig. 2). It seems that this morphological gradient is related to the differentiation between faunivores and consumers of plant matter (herbivores and omnivores). Whereas faunivore rodents, with more elliptical and tight outlines, were grouped around the highest values of this axis, other murines were placed mostly on the negative part of the axis, presenting rectangular and wide outlines. The presence of broad molars is characteristic for taxa including a plant component in their diets [40]. On the other hand, faunivore genera may show a tendency to reduce occlusal surface in their molars, probably due to the development of robust incisors [10]. Furthermore, the tendency towards buccolingual compression of the M1 shown by faunivore murines agrees with the observed general trend in Carnivora, which present narrowed molars by aligning their cusps anteroposteriorly as a specialization for shearing or slicing [41].

The second canonical axis (CV2) described a variation from more pronounced cusps in an undulated outline on the positive side of the axis towards more symmetrical and rounded outlines on the negative side (Fig. 2). These shapes are congruent with previous works on Murinae rodents, which associated a slender asymmetrical outline with omnivory, which are at the positive boundary of the CV2 axis, and a broader and symmetrical one with herbivory, which is at the negative edge of our CV2 axis [31], [42]. This dental feature should be functionally related to a relative increase in occlusal surface and increased grinding efficiency [43], which might be associated with cusp broadening and a progressive increase in enamel surface with tooth wearing. Moreover, the morphological trend of this CV2 represents the variation across the generalist/specialist gradient. This contrast of differentiated outline between generalist and specialist was already highlighted by Renaud et al. [9] in a geometric morphometric study of murine mandibles. The results in that study emphasize the role of ecological diversification in determining the rhythm of morphological evolution, indicating that lower morphological divergences only involve omnivores, instead large morphological divergences involving the specialist taxa, which correspond to diverse feeding behaviours diverging from the omnivorous diet.

Despite the congruence between morphology and feeding habits, the high morphological variability contained within the genera studied as well as the interspecific differences in ecology within each genus are responsible for the existence of overlapping areas among the different dietary categories. Furthermore, the evolutionary history of the genera studied with changes in feeding habits among related lineages, is also likely related to the presence of this overlap. For example, the omnivore *Margaretamys*, which is placed in the overlapping area between the morphospaces defined by faunivore and omnivore murines, is closely related to the faunivore genera *Echiothrix*, *Melasmothrix* and *Tateomys* [44], [45]. Likewise, *Micromys* (omnivore) and *Millardia* (herbivore), which also occur in this overlapping area, configure a monophyletic clade with the herbivore *Pogonomys* [44], [45].

Finally, we did not find a statistically significant relation between size and CV1 (r = 0.023; p = 0.842) nor CV2 (r = 0.028; p = 0.072). Furthermore, there was not significant result in the ANOVA of size and feeding habits in murine rodents. This could be explained because the size factor is part of a more complex pattern linked to the development of the whole dental row and the patterning cascade model of development of the molars, in which are implied the size of other dental elements [21], [46] are involved.

### Dietary Inference in Extinct Murinae

The discriminant functions for the distinction among different diets in extant murines correctly classified 84.2% of the genera. Percentages of well-classified genera are similarly distributed in each dietary category (table 2). Genera that have been classified into an erroneous dietary group are those included in the overlapping area of the CVA plot (Fig. 2).

**Table 2:** Percentages of well-classified genera in the discriminant analysis.

	Pronosticated group		
Diet	Herbivore	Omnivore	Faunivore
Herbivore	81.80	18.20	0.00
Omnivore	15.00	82.50	2.50
Faunivore	7.10	14.30	78.60

Our dietary reconstructions for the extinct murine genera from the Iberoccitanian Upper Miocene indicated an omnivore dietary preference for most of them (table 3), with the exception of *Stephanomys*, which was classified as herbivore. These results, were therefore roughly consistent with previous studies focusing on the evolution of morphological variation in *Occitanomys* [31] or *Progonomys* [43], which also indicated omnivore preferences for these extinct genera. The reconstructed herbivore diet for *Stephanomys* agrees with the classical assumptions for this genus. Several studies indicate a particular preference of *Stephanomys* for feeding on grass, due to the presence of morphological features in the dental pattern associated with the stephanodonty [31], [43], [47], [40]. This dental pattern is characterised by the presence of longitudinal ridges between molar cusps as well as by broad and symmetrical molars [48], [39], [49].

**Table 3:** Discriminant analysis results for the 85 genera considered.

		Probability of belonging to		
Genus	Diet	Herbivore	Omnivore	Faunivore
*Abditomys*	Herbivore	0.979	0.017	0.004
*Chiropodomys*	Herbivore	0.891	0.108	0.001
*Golunda*	Herbivore	0.994	0.006	0.000
*Hadromys*	Herbivore	0.989	0.011	0.000
*Haeromys*	Herbivore	0.911	0.088	0.002
*Hapalomys*	Herbivore	1.000	0.000	0.000
*Hyomys*	Herbivore	0.800	0.200	0.000
*Kadarsanomys*	Herbivore	0.976	0.018	0.006
*Leporillus*	Herbivore	0.357	0.434*	0.208
*Mallomys*	Herbivore	0.849	0.150	0.000
*Mastacomys*	Herbivore	0.993	0.007	0.001
*Melomys*	Herbivore	0.957	0.043	0.000
*Millardia*	Herbivore	0.577	0.423	0.000
*Papagonomys*	Herbivore	0.990	0.007	0.002
*Pelomys*	Herbivore	0.364	0.636*	0.000
*Phloeomys*	Herbivore	0.888	0.109	0.003
*Pogonomys*	Herbivore	0.911	0.089	0.000
*Solomys*	Herbivore	0.927	0.066	0.006
*Spelaeomys*	Herbivore	0.376	0.624*	0.000
*Uromys*	Herbivore	0.359	0.507*	0.134
*Vandeluria*	Herbivore	0.522	0.450	0.028
*Aethomys*	Omnivore	0.050	0.950	0.000
*Anisomys*	Omnivore	0.641*	0.334	0.025
*Apodemus*	Omnivore	0.315	0.685	0.000
*Apomys*	Omnivore	0.017	0.551	0.432
*Arvicanthis*	Omnivore	0.026	0.973	0.001
*Bandicota*	Omnivore	0.092	0.906	0.002
*Bunomys*	Omnivore	0.018	0.982	0.000
*Coccymys*	Omnivore	0.746*	0.210	0.044
*Crateromys*	Omnivore	0.040	0.960	0.000
*Dasymys*	Omnivore	0.918	0.082	0.000
*Eropeplus*	Omnivore	0.006	0.951	0.043
*Grammomys*	Omnivore	0.088	0.909	0.003
*Hybomys*	Omnivore	0.496	0.503	0.001
*Hylomyscus*	Omnivore	0.455	0.537	0.007
*Leggadina*	Omnivore	0.013	0.985	0.002
*Lemniscomys*	Omnivore	0.010	0.989	0.000
*Lenomys*	Omnivore	0.073	0.927	0.000
*Leopoldomys*	Omnivore	0.042	0.958	0.000
*Lorentzimys*	Omnivore	0.016	0.984	0.000
*Malacomys*	Omnivore	0.305	0.681	0.014
*Margaretamys*	Omnivore	0.021	0.587	0.392
*Mastomys*	Omnivore	0.267	0.725	0.008
*Maxomys*	Omnivore	0.041	0.957	0.001
*Micromys*	Omnivore	0.403	0.596	0.002
*Mus*	Omnivore	0.003	0.997	0.000
*Nivivemter*	Omnivore	0.033	0.104	0.863
*Notomys*	Omnivore	0.078	0.922	0.000
*Oenomys*	Omnivore	0.298	0.702	0.000
*Pitecheir*	Omnivore	0.340	0.659	0.000
*Praomys*	Omnivore	0.006	0.994	0.000
*Pseudohydromys*	Omnivore	0.000	0.998	0.002
*Pseudomys*	Omnivore	0.258	0.741	0.001
*Rattus*	Omnivore	0.024	0.968	0.008
*Rhabdomys*	Omnivore	0.150	0.839	0.011
*Stochomys*	Omnivore	0.450	0.544	0.006
*Sundamys*	Omnivore	0.058	0.909	0.033
*Thallomys*	Omnivore	0.002	0.967	0.031
*Thammomys*	Omnivore	0.599	0.400	0.001
*Tokudaia*	Omnivore	0.228	0.744	0.028
*Zelotomys*	Omnivore	0.020	0.956	0.024
*Zyzomys*	Omnivore	0.001	0.999	0.000
*Archboldomys*	Faunivore	0.000	0.000	1.000
*Colomys*	Faunivore	0.360	0.578	0.062
*Crossomys*	Faunivore	0.000	0.000	1.000
*Crunomys*	Faunivore	0.040	0.819	0.141
*Chrotomys*	Faunivore	0.000	0.000	0.999
*Echiothrix*	Faunivore	0.766*	0.149	0.086
*Hydromys*	Faunivore	0.001	0.001	0.998
*Leptomys*	Faunivore	0.000	0.001	0.999
*Melasmothrix*	Faunivore	0.000	0.000	1.000
*Parahydromys*	Faunivore	0.000	0.000	1.000
*Paulamys*	Faunivore	0.000	0.000	1.000
*Rhynchomys*	Faunivore	0.014	0.033	0.953
*Sommeromys*	Faunivore	0.005	0.066	0.929
*Tateomys*	Faunivore	0.000	0.000	1.000
*Anthracomys*		0.000	0.895	0.105
*Castillomys*		0.225	0.775	0.000
*Castromys*		0.093	0.906	0.001
*Huerzelerimys*		0.150	0.850	0.000
*Occitanomys*		0.173	0.827	0.000
*Paraethomys*		0.112	0.887	0.000
*Progonomys*		0.059	0.941	0.000
*Rhagapodemus*		0.001	0.998	0.000
*Stephanomys*		0.896	0.104	0.001

The table shows the values of high probability for each genus in the three different dietary categories.

* indicates erroneous classification in the extant genera; Extinct genera are shown in grey.

When compared to modern faunas from areas under tropical or subtropical climate regimes, such as the ones inferred for the Iberoccitanian region during the Late Miocene [50], [51], the dominance of omnivorous taxa in our results appears to point to subtropical climatic conditions [52], in which herbivore taxa are very scarce. This agrees with the palaeoclimatic data provided for herpetofaunas [53], [54] and plants [55]–[57]. Additionally, this predominance of omnivorous taxa might be also associated with the arrival of murines to southwestern Europe. In general, species capable of exploiting a wide variety of resources tend to be more widespread than the more specialized ones [58], [59], and are probably responsible for most of the large dispersals. This is the case for *Progonomys*, the first murine found in Europe [60], as well as for most of the other genera from the Iberoccitanian region [61]. Only *Stephanomys* appears to be the result of strong directional selection towards herbivory [43], [62], presumably imposed by aridification in the Iberoccitanian region around 7 ma [53].

Finally, since we included all the species variability of one genus within an average M1 outline for the genus, one can assume the presence of more specialist species within the genera we considered as omnivores. For example, [40] evidenced the diversity of feeding habits of species within *Occitanomys*; *O. adroveri* and *O. sondaari* were specialised in feeding on grass whereas *O. alcalai* was inferred as a non grass feeder. As a consequence of this interspecific variability there can be differences between our results and those from studies focusing on the species level [7], [40].

## Conclusions

This is the first time to our knowledge, that geometric morphometric comparison of the outlines of the first upper molar in the highly diverse extant murines has enabled the inference of ecological preferences in diet based on dental morphology. In the morphometric space described, all dietary groups were significantly distinguished from each other. Furthermore, based on the data of extant murines, we were able to infer the dietary preferences of nine extinct genera of murine rodents, which have been described at the Iberoccitanian fossil sites from the Upper Miocene.

Finally, Elliptic Fourier Analysis has been shown to constitute an interesting tool for inferring ecological preferences in extinct rodents, which are mostly recorded as isolated teeth in the fossil sites. The results of our study open up possibilities for establishing new comparisons in other mammalian groups, and for making ecological inferences of extinct taxa based upon information referring to their extant relatives. Furthermore, with the increasing development of phylogenetic studies, such inferences would help to map the evolutionary history of feeding habits.

## Supporting Information

Appendix S1 Dietary preferences of extant murine genus and references. Diet determination in literature resumed as 1: field data, feeding trials, stomach morphology or bibliographic compilation; 2: stomach content or faecal pellet analyses.
(PDF)

Appendix S2 Collection number and references of the extant and extinct murine rodent used in this work. *indicates the specimens for which we take the photograph in the Musée National d’Histoire Naturelle (Paris); Grey fonts for extinct genera.
(PDF)

Appendix S3 Estimation of the information content in each harmonic based on its amplitude. The amplitudes were cumulated over the total range of harmonics and the information brought by each harmonic was estimated as the percentage of the sum of all harmonic amplitudes. In our case each of the nine first harmonics increased the amount of shape information up to 97% of the total information, meanwhile the subsequent harmonics provided almost no further relevant shape information (Fig. S1).
(PDF)

Appendix S4 Fourier Components and size. Values estimated for each extant and extinct genus considered in this works after EFA.
(PDF)
