# Supplementary material for: Dietary Ecology of Murinae (Muridae, Rodentia): A Geometric Morphometric Approach
Source: PLoS One. 2013 Nov 13;8(11):e79080. doi: 10.1371/journal.pone.0079080 (PMC3827291; doi:10.1371/journal.pone.0079080)
Supplement: Appendix S1 — Dietary preferences of extant murine genus and references. Diet determination in literature resumed as 1: field data, feeding trials, stomach morphology or bibliographic compilation; 2: stomach content or faecal pellet analyses. (PDF) [file pone.0079080.s001.pdf]

Appendix S1. Dietary preferences of extant murine genus and references. Diet determination in literature resumed as 1: field data, feeding trials, stomach morphology or bibliographic compilation; 2: stomach content or faecal pellet analyses.

Genus	Diet	Diet determination	References
Abditomys	Herbivore	1	Musser [1]; Heaney [2]
Aethomys	Omnivore	1, 2	Martin [3]
Anisomys	Omnivore	1	Martin [3]
Apodemus	Omnivore	1, 2	Martin [3]
Apomys	Omnivore	2	Martin [3]
Archboldomys	Faunivore	1, 2	Martin [3]
Arvicanthis	Omnivore	2	Martin [3]
Bandicota	Omnivore	2	Martin [3]
Bunomys	Omnivore	1, 2	Suripto and Aganto Seno [4] ; Musser and Durden [5]; Musser and Lunde [6]; Martin [3]
Chiropodomys	Herbivore	1	Musser [1]
Chrotomys	Faunivore	1, 2	Martin [3]
Coccymys	Omnivore	1, 2	Musser and Lunde [6]; Martin [3]
Colomys	Faunivore	1, 2	Martin [3]
Crateromys	Omnivore	1, 2	Martin [3]; Aplin and Helgen [7]
Crossomys	Faunivore	1	Helgen and Helgen [8]; Musser and Lunde [6]
Crunomys	Faunivore	1, 2	Musser and Durden [5]
Dasymys	Omnivore	1	Nowak [9]; Gomes Rodrigues et al. [9]
Echiothrix	Faunivore	1	Musser [10]
Eropeplus	Omnivore	1, 2	Musser and Durden [5]; Musser and Lunde [6]; Martin [3]
Golunda	Herbivore	1	Nowak [11]; Gomes

			Rodrigues et al. [9]
Grammomys	Omnivore	1,	Gomes Rodrigues et al. [9]; Martin [3]
Hadromys	Herbivore	2	Nowak [9]; Chaimanee and Jaeger [12]
Haeromys	Herbivore	1	Musser and Durden [5]; Musser and Lunde [6]
Hapalomys	Herbivore	1	Musser [13]; Martin [3]
Hybomys	Omnivore	1, 2	Gomes Rodrigues et al. [9]; Martin [3]; Fa and Purvis [14]
Hydromys	Faunivore	2	Rowe et al. [15]; Helgen and Helgen [8]; Musser and Lunde [6]; Martin [3]
Hylomyscus	Omnivore	1	Gomes Rodrigues et al. [9]; Martin [3]
Hyomys	Herbivore	1	Samuels [16]; Martin [3]
Kadarsanomys	Herbivore	1	Musser [13]
Leggadina	Omnivore	1, 2	Nowak [9]; Read [17]; Kelt et al. [18]
Lemniscomys	Omnivore	1, 2	Martin [3]
Lenomys	Omnivore	1	Musser and Durden [5]; Musser and Lunde [6]; Martin [3]
Leopoldamys	Omnivore	1, 2	Charles and Ang [19] ; Martin [3]
Leporillus	Herbivore	1	Martin [3]
Leptomys	Faunivore	1, 2	Helgen and Helgen [8]; Musser and Lunde [6];

			Martin [3]; Musser et al. [20]
Lorentzimys	Omnivore	2	Martin [3]
Malacomys	Omnivore	1	Martin [3]
Mallomys	Herbivore	1	Rowe et al. [15]; Samuels [16]; Aplin and Helgen [21]; Martin [3]
Margaretamys	Omnivore	1	Musser and Durden [5]; Musser and Lunde [6]; Martin [3]
Mastacomys	Herbivore	1	Rowe et al. [15]; Bezzobs and Sanson [22]
Mastomys	Omnivore	2	Martin [3]
Maxomys	Omnivore	1, 2	Charles and Ang [19] ; Musser and Durden [5]; Musser and Lunde [6]; Martin [3]
Melasmothrix	Faunivore	1	Musser and Durden [5]; Musser et al. [23]; Samuels [16]; Martin [3]
Melomys	Herbivore	1	Martin [3]
Micromys	Omnivore	2	Martin [3]
Millardia	Herbivore	2	Martin [3]
Mus	Omnivore	1, 2	Martin [3]
Niviventer	Omnivore	1, 2	Charles and Ang [19] ; Renaud et al. [24]; Wu and Yu [25]
Notomys	Omnivore	1, 2	Martin [3]
Oenomys	Omnivore	1	Gomes Rodrigues et al. [9]; Martin [3]; Fa and Purvis [14]
Papagomys	Herbivore	1	Musser [14]
Parahydromys	Faunivore	2	Helgen and

			Helgen [8]; Musser and Lunde [6]; Martin [3]
Paulamys	Faunivore	1	Musser et al. [26]
Pelomys	Herbivore	1, 2	Gomes Rodrigues et al. [9]; Martin [3]; Fa and Purvis [14]
Phloeomys	Herbivore	1	Musser and Heaney [27]; Samuels [16]; Martin [3]
Pithecheir	Omnivore	1	Nowak [9]
Pogonomys	Herbivore	1, 2	Martin [3]
Praomys	Omnivore	1	Gomes Rodrigues et al. [9]; Martin [3]; Fa and Purvis [14]
Pseudohydromys	Omnivore	2	Musser and Lunde [6]; Martin [3]
Pseudomys	Omnivore	1, 2	Martin [3]; Casanovas-Vilar et al. [28]
Rattus	Omnivore	1, 2	Samuels [16]; Martin [3]
Rhabdomys	Omnivore	1, 2	Martin [3]; Fa and Purvis [14]
Rhynchomys	Faunivore	1, 2	Rowe et al. [15]; Helgen and Helgen [8]; Musser and Lunde [6]; Samuels [16]; Martin [3] ; Balete et al. [29]
Solomys	Herbivore	1	Nowak [9]
Sommeromys	Faunivore	1, 2	Musser and Durden, (2002)
Spelaeomys	Herbivore	1	Musser [14]
Stochomys	Omnivore	1, 2	Gomes Rodrigues et al. [9]; Fa and Purvis [14]
Sundamys	Omnivore	1, 2	Charles and

			Ang [19] ; Renaud et al. [24]
Tateomys	Faunivore	1	Musser [30]; Samuels [16]; Martin [3]
Thallomys	Omnivore	1, 3	Nowak [11]; Gomes Rodrigues et al. [9]; Martin [3]; Meyera et al. [31]; Coleman and Downs [32]
Thamnomys	Omnivore	1, 2	Martin [3]; [14]
Tokudaia	Omnivore	1	Renaud et al. [24]
Uromys	Herbivore	1, 2	Nowak [9]; Samuels [16]
Vandeleuria	Herbivore	1	Nowak [9]
Zelotomys	Omnivore	2	Martin [3]
Zyzomys	Omnivore	2	Martin [3]

References:

1. Musser GG (1982) Results of the Archbold Expeditions .No. 107. A New Genus of Arboreal Rat from Luzon Island in the Philippines. American Museum Novitates 2730: 1-24.
2. Heaney L (2008) Abditomys latidens. In: IUCN 2012 IUCN Red List of Threatened Species Version 20121 wwwiucnredlistorg Downloaded on July 2012.
3. Martin SA (2010) Dental adaptation in murine rodents (Muridae): assessing mechanical predictions. Florida: The Florida State University. 73 p.
4. Suripto BA, Aganto Seno SJPTII, Journal of Plant Protection), 8(2002). (2002) Jenis-Jenis Tikus (Rodentia: Muridae) Dan Pakan Alaminya Di Daerah Pertanian Sekitar Hutan Di Kabupaten Banggai, Sulawesi Tengah The Species Of Rats (Rodentia: Muridae) And Their Foods In Agricultural Area In Surounding. Jurnal Perlindungan Tanaman Indonesia (Indonesian, Journal of Plant Protection) 8.
5. Musser GG, Durden LA (2002) Sulawesi rodents: Description of a new genus and species of Murinae (Muridae, Rodentia) and its parasitic new species of sucking louse (Insecta, Anoplura) (American Museum novitates). American Museum Novitates 3368: 1-50.
6. Musser GG, Lunde DP (2009) Systematic Reviews of New Guinea Coccymys and "Melomys" Albidens (Muridae, Murinae) with Descriptions of New Taxa. Bulletin of the American Museum of Natural History 329: 1-139.
7. Aplin KP, Helgen KM (2010) Quaternary murid rodents of Timor Part I: New material of Coryphomys buehleri Schaub, 1937, and description of a second species of the Genus. Bulletin of the American Museum of Natural History, 1-80. Bulletin of the American Museum of Natual History 341: 1-80.
8. Helgen KM, Helgen LE (2009) Chapter 8. Biodiversity and Biogeography of the Moss-mice of New Guinea: A Taxonomic Revision of Pseudohydromys (Muridae: Murinae). Bulletin of the American Museum of Natural History 331: 230-313.
9. Gomes Rodrigues H, Merceron G, Viriot L (2009) Dental microwear patterns of extant and extinct Muridae (Rodentia, Mammalia): ecological implications. Naturwissenschaften 96: 537-542.
10. Musser GG (1990) Sulawesi Rodents: Species Traits and ChromosomesofHaeromys minahassaeand Echiothrixleucura(Muridae:Murinae). American Museum Novitates 2989: 1-20.
11. Nowak RM, editor (1999) Walker's Mammals of the World: The John Hopkins University Press.
12. Chaimanee Y, Jaeger J-J (2000) Occurrence of *Hadromys humei* (Rodentia: Muridae) during the Pleistocene in Thailand. Journal of Mammalogy 81: 659-665.
13. Musser GG (1982) Results of the Archbold Expeditions. No. 108. The Definition of Apomys, a Native Rat of the Philippine Islands. American Museum Novitates 2746: 1-44.
14. Fa JE, Purvis A (1997) Body size, diet and population density in Afrotropical forest mammals: a comparison with neotropical species. Journal of Animal Ecology 66: 98-112.
15. Rowe KC, Reno ML, Richmond DM, Adkins RM, Steppan SJ (2008) Pliocene colonization and adaptive radiations in Australia and New Guinea (Sahul):

Multilocus systematics of the old endemic rodents (Muroidea: Murinae). Molecular Phylogenetics and Evolution 47: 84-101.

16. Samuels JX (2009) Cranial morphology and dietary habits of rodents. Zoological Journal of the Linnean Society 156: 864-888.
17. Read DG (1984) Habitat preference and diet of Leggadina forresti in western New South Wales. Australian Mammalogy 7: 215-217.
18. Kelt DA, Brown JH, Heske EJ, Marquet PA, Morton SR, et al. (1996) Community structure of desert small mammals: comparisons across four continents. Ecology 77: 746-761.
19. Charles JK, Ang BB (2010) Non-volant small mammal community responses to fragmentation of kerangas forests in Brunei Darussalam. Biodiversity and Conservation 19: 543-561.
20. Musser GG, Helgen KM, Lunde DP (2008) Systematic review of New Guinea *Leptomys* (Muridae, Murinaei): with descriptions of two new species. American Museum Novitates 3624: 1-60.
21. Aplin KP, Helgen KM (2010) Quaternary Murid Rodents of Timor Part I: New Material of Coryphomys buehleri Schaub, 1937, and Description of a Second Species of the Genus. Bulletin of the American Museum of Natural History: 1-80.
22. Bezzobs T, Sanson G (1997) The effects of plant and tooth structure on intake and digestibility in two small mammalian herbivores. Physiological zoology 70: 338.
23. Musser G, Lunde D, Ruedas L (2008) Melasmothrix naso. IUCN 2012 IUCN Red List of Threatened Species Version 20122 wwwiucnredlistorg Downloaded on October 2012.
24. Renaud S, Chevret P, Michaux J (2007) Morphological vs. molecular evolution: ecology and phylogeny both shape the mandible of rodents. Zoologica Scripta 36: 525-535.
25. Wu H-Y, Yu H-T (2004) Spatial Organization of a Forest-Dwelling Murid Rodent, *Niviventer coxingi*, in Subtropical Central Taiwan. Zoological Studies 43: 612-621.
26. Musser GG, van de Weerd A, Strasser E (1986) Paulamys, a replacement name for Floresomys Musser 1981 (Muridae), and new material of that taxon from Flores, Indonesia. Am Museum Novitates 2850: 1-10.
27. Musser GG, Heaney LR (1992) Philippine Rodents: Definitions of Tarsomys and Limnomys plus a preliminary assessment of phylogenetic patterns among native philippine murines (Murinae, Muridae). Bulletin of the American Museum of Natural History 211: 1-144.
28. Casanovas-Vilar I, Van Dam jA, Moyà-Solà S, Rook L (2011) Late Miocene insular mice from the Tusco-Sardinian palaeobioprovince provide new insights on the palaeoecology of the *Oreopithecus* faunas. Journal of Human Evolution 61: 42-49.
29. Balete DS, Rickart EA, Rosell-Ambal RGB, Jansa S, Heaney LR (2007) Descriptions of two new species of *Rhynchomys* Thomas (Rodentia: Muridae: Murinae) from Luzon Island, Philippines. Journal of Mammalogy 88: 287-301.
30. Musser GG (1969) Results of the Archbold expeditions. No. 91. A new genus and species of murid rodent from Celebes, with a discussion of its relationships. American Museum Novitates 2384: 1-41.
31. Meyera J, Raudnitschkab D, Steinhauserc J, Jeltschc F, Brandla R (2008) Biology and ecology of Thallomys nigricauda (Rodentia, Muridae) in the Thornveld

savannah of South Africa. Mammalian Biology - Zeitschrift für Säugetierkunde 73: 111-118.
32. Coleman JC, Downs CT (2009) Variation in urine concentrating ability and water balance of the Black-tailed Tree Rat Thallomys nigricauda, along an aridity gradient. Comparative Biochemistry and Physiology Part A: Molecular & Integrative Physiology 154: 508-513.
