# Supplementary material for: Dietary Ecology of Murinae (Muridae, Rodentia): A Geometric Morphometric Approach
Source: PLoS One. 2013 Nov 13;8(11):e79080. doi: 10.1371/journal.pone.0079080 (PMC3827291; doi:10.1371/journal.pone.0079080)
Supplement: Appendix S2 — Collection number and references of the extant and extinct murine rodent used in this work. *indicates the specimens for which we take the photograph in the Musée National d’Histoire Naturelle (Paris); Grey fonts for extinct genera. (PDF) [file pone.0079080.s002.pdf]

Appendix S2. Collection number and references of the extant and extinct murine rodent used in this work. * indicates the specimens for which we take the photograph in the Musée National d'Histoire Naturelle (Paris); Grey fonts for extinct genera.

Species	Reference	Fossil site	Collection number
Abditomys sp.	Musser and Heaney [1]		USNM 357244
Aethomys hindei	*		MNHN 1999 177
Aethomys namanquesis	*		MNHN 1964 57
Aethomys nigeriae	*		MNHN 1996 2239
Anisomys imitator	Misonne [2]		BMNH 471310
Apodemus agrarius	*		MNHN BL6 1134
Apodemus sylvaticus	*		MNHN 1994 2667
Apodemus sylvaticus	*		MNHN 1994 2668
Apodemus sylvaticus	*		MNHN 1994 2671
Apodemus sylvaticus	*		MNHN 1994 2679
Apodemus sylvaticus	*		MNHN 1994 2681
Apodemus sylvaticus	*		MNHN 1994 945
Apomys sp.	Musser and Heaney [1]		12971 CLM1-3
Archboldomys sp.	Musser and Heaney [1]		FMNH 95122
Arvicanthis niloticus	*		MNHN 2007 217
Bandicota indica	*		MNHN 1986 366
Bunomys chysocomus	Durden and Musser [3]		MZB 12185
Bunomys prolatus	Durden and Musser [3]		MZB 12190
Chiropodomys calamianensis	Tate [4]		AM109999
Chiropodomys gliroides	*		MNHN 1981 283
Chrotomys sp.	Musser and Heaney [1]		AMNH185138
Chrotomys sp.	Musser and Heaney [1]		FMNH 62279
Chrotomys sp.	Musser and Heaney [1]		MMNH 12972
Coccymys ruemmleri	Musser and Lunde [5]		AMNH 192737
Colomys goslingi	Misonne [2]		MRAC 21953
Crateromys sehadenbergi	*		MNHN 1897 414
Crateromys sp.	Musser and Heaney [1]		USNM 102546
Crunomys melanius	Musser and Durden [6]		AMNH 224316
Crunomys sp.	Musser and Heaney [1]		AMNH 242102
Dasymys incomitus	*		MNHN 1970 563 3977
Dasymys incomptus	*		MNHN 1982 1037
Dasymys rufulus	*		MNHN 2004 1091
Echiothrix leucura	Musser [7]		AMNH 225681
Echiothrix leucura	Tate [4]		101248
Eropeplus canus	Misonne [2]		USNM 3336
Eropeplus canus	Tate [4]		AM2601
Golunda ellioti	*		MNHN 1999 17
Grammomys butingi	*		MNHN 1980 270
Grammomys caniceps	*		MNHN 1986 1091
Grammomys dolichirus	*		MNHN 2005 6175
Grammomys dolichurus	*		MNHN 2006 602
Grammomys dolichurus	*		MNHN 2006 603
Grammomys gazellae	*		MNHN 1982 528
Grammomys macmillani	*		MNHN 2000 28
Grammomys macmillani	*		MNHN 2000 29
Grammomys rutilans	*		MNHN 1966 244
Grammomys rutilans	*		MNHN 1996 2221
Grammomys sudaster	*		MNHN 1996 680
Hadromys humei	Misonne [2]		BMNH 217871
Hadromys humei	Musser [8]		FMNH 76567
Haeromys minahassae	Musser [7]		AMNH 226048

Hapalomys longicaudatus	*	MNHN 1977 248
Hapalomys longicaudatus	Tate [4]	AM54754
Heimyscus fumosus	*	MNHN 2008 244
Hybomys trivirgatus	*	MNHN 1979 420
Hybomys univittatus	*	MNHN 1963 210
Hydromys habbema	Musser and Heaney [1]	AMNH 110057
Hydromys hussoni	Helgen [9]	
Hylomyscus waltervercheyeni	*	MNHN 2007 754
Hyomys alleni	*	MNHN 1991 2128
Hyomys meeki	Tate [4]	AM79781
Kadarsanomys sodyi	Musser [12]	RMNH14103
Leggadina delicatulus	Misonne [2]	BMNH 35742
Leggadina hermannsburgensis	*	MNHN 1971 543
Leggadina lakedownensis	Cooper et al. [10]	M16906
Lemniscomys barbarus	*	MNHN 1975 117
Lemniscomys bellieri	*	MNHN 2006 208
Lemniscomys griselda	*	MNHN 1889 190
Lemniscomys linulus	*	MNHN 2004 1063
Lemniscomys macculus	*	MNHN 1992 1549
Lemniscomys rosalia	*	MNHN 1996 569
Lemniscomys zebra	*	MNHN 2004 135
Lenomys meyeri	Misonne [2]	BMNH 991019
Lenomys meyeri	Tate [4]	AM1011125
Lenothrix canus	*	MNHN 1977 246
Leopoldomys sabanus	*	MNHN 1977 377
Leporillus apicalis	*	MNHN 1882 2173
Leptomys sp.	Musser and Heaney [1]	AMNH 105793
Lorentzimys nouhuysi	Misonne [2]	BMNH 53329
Malacomys edwardsi	*	MNHN 2008 86
Malacomys longipes	*	MNHN 1995 1217
Malacomys lukolelae	*	MNHN 1991 869
Mallomys rothschildi	Misonne [2]	BMNH 501781
Mallomys rothschildi	Tate [4]	AM104154
Margaretamys beccarii	Musser [12]	AMNH 224061
Margaretamys beccarii	Musser [12]	AMNH 224064
Margaretamys elegans	Musser [12]	AMNH 225143
Margaretamys parvus	Musser [12]	AMNH 226068
Mastacomys fuscus	*	MNHN A2471
Mastomys coucha	*	MNHN 1971 509
Maxomys inas	*	MNHN 1977 206
Maxomys wattsi	Durden and Musser [3]	MZB 12155
Melasmothrix naso	Misonne [2]	M12 4.4
Melomys levipes	*	MNHN 1985 1943
Melomys lorentzii	*	MNHN 1991 647
Melomys rubex	*	MNHN 1995 1560
Melomys sp.	Musser [11]	
Micromys minutus	*	MNHN 1932 4543
Millardia meltada	*	MNHN 1957 556
Mus booduga	*	MNHN 1968 5
Mus bufo	*	MNHN 1966 2215
Mus caroli	*	MNHN 1981 829
Mus cervicolor	*	MNHN 1981 839
Mus confucianus	*	MNHN 2007 218
Mus cookii	*	MNHN 1981 824

Mus cypriacus	*	MNHN 2005 834
Mus dunni	*	MNHN 1968 15
Mus famulus	*	MNHN 200 252
Mus fragicauda	*	MNHN 1999 1067
Mus fulvidiventris	*	MNHN 1973 321
Mus goundae	*	MNHN 1971 402
Mus gratus	*	MNHN 2005 686
Mus haussa	*	MNHN 1969 31
Mus mahomet	*	MNHN 1972 248
Mus mattheyi	*	MNHN 1972 732
Mus minutoides	*	MNHN 1970 185
Mus musculoides	*	MNHN 1977 471
Mus musculus	*	MNHN 1933 1871
Mus musculus	*	MNHN 1933 1893
Mus musculus	*	MNHN 1942 353
Mus musculus	*	MNHN 1947 854
Mus musculus	*	MNHN 1947 855
Mus musculus	*	MNHN 1953 811
Mus musculus	*	MNHN 1953 812
Mus musculus	*	MNHN 1956 613
Mus musculus	*	MNHN 1957 1323
Mus musculus	*	MNHN 1957 369
Mus musculus	*	MNHN 1957 371
Mus musculus	*	MNHN 1957 373
Mus musculus	*	MNHN 1957 380
Mus musculus	*	MNHN 1957 545
Mus musculus	*	MNHN 1958 358
Mus musculus	*	MNHN 1958 359
Mus musculus	*	MNHN 1961 1064
Mus musculus	*	MNHN 1961 1065
Mus musculus	*	MNHN 1969 101
Mus musculus	*	MNHN 1969 102
Mus musculus	*	MNHN 1969 103
Mus musculus	*	MNHN 1970 334
Mus musculus	*	MNHN 1973 344
Mus musculus	*	MNHN 1980 207
Mus musculus	*	MNHN 1991 1185
Mus musculus	*	MNHN 2000 253
Mus Nannomys	*	MNHN 1970 99
Mus natalensis	*	MNHN A7463
Mus oubanguii	*	MNHN 1971 446
Mus praetextus	*	MNHN 1991 1166
Mus Pyromys	*	MNHN 1997 2067
Mus setulosus	*	MNHN 1964 12
Mus shortidgei	*	MNHN 1981 825
Mus spretus	*	MNHN 1980 357
Mus spretus	*	MNHN 1980 417
Mus tenellus	*	MNHN 1977 27
Mus triton	*	MNHN 1996 414
Nivivemter coxingi	*	MNHN 1874 634
Niviventer cremoriventer	Musser [12]	AMNH 103579
Niviventer fulvescens	*	MNHN 1981 1292
Notomys alexis	*	MNHN 2001 471
Notomys mitchelli	*	MNHN 1882 2172
Oenomys hypoxanthus	*	MNHN 1995 3002
Papagonomys verhoeyeni	Misonne [2]	
Parahydromys asper	Helgen [9]	

Paulamys sp.	Kitchener et al. [13]	WAMM32000
Pelomys fallax	*	MNHN 1952 881
Phloeomys cumingi	*	MNHN 1962 2564
Phloeomys pallidus	Misonne [2]	BMNH 97317
Phloeomys sp.	Musser and Heaney [1]	AMNH242103
Pitecheir melanurus	*	MNHN 1894 1663
Pogonomys fergussoniensis	Misonne [2]	BMNH 501176
Pogonomys forbesi	Tate [4]	79828
Pogonomys lepidus	Tate [4]	AM104202
Pogonomys macrourus	*	MNHN 2005 823
Pogonomys sylvestris	Tate [4]	AM79757
Praomys daltoni	*	MNHN 1982 697
Praomys daltoni	*	MNHN 1992 1573
Praomys derooi	*	MNHN 1964 359
Praomys tullbergi	*	MNHN 1992 1438
Praomys yemenni	*	MNHN 1996 301
Pseudohydromys patriciae	Helgen and Helgen [14]	AM M26991
Pseudomys australis	*	MNHN 1995 1561
Rattus andersoni	*	MNHN 1896 2098
Rattus annandalei	*	MNHN 1981 250
Rattus argentiventer	*	MNHN 1969 150
Rattus confucianus	*	MNHN 1929 464
Rattus exiguus	*	MNHN 1981 159
Rattus exulans	*	MNHN 1991 1191
Rattus exulans	*	MNHN 2009 117
Rattus fuscipes	*	MNHN 1846 1364
Rattus koratensis	*	MNHN 1990 540
Rattus niobe	Tate [4]	AM104289
Rattus nitidus	*	MNHN 1899 122
Rattus norvergicus	*	MNHN 1862 472A
Rattus norvergicus	*	MNHN 1873 234
Rattus norvergicus	*	MNHN 1874 633
Rattus norvergicus	*	MNHN 1903 35
Rattus norvergicus	*	MNHN 1933 2110
Rattus norvergicus	*	MNHN 1986 1204
Rattus norvergicus	*	MNHN 1995 3266
Rattus rajah	*	MNHN 1996 2292
Rattus rattus	*	MNHN 1902 1235
Rattus rattus	*	MNHN 1902 511
Rattus rattus	*	MNHN 1911 2352A
Rattus rattus	*	MNHN 1952 512
Rattus rattus	*	MNHN 1962 1841
Rattus rattus	*	MNHN 1994 840
Rattus rattus	*	MNHN 2007 1099
Rattus rattus	*	MNHN 2007 311
Rattus sikkimensis	*	MNHN 1995 2833
Rattus sladeni	*	MNHN 1997 79
Rattus tanezumi	*	MNHN 1874 616
Rattus tiomanicus	*	MNHN 1981 273
Rattus turkestanicus	*	MNHN 1991 1319
Rattus verecundus	Tate [4]	AM104277
Rhabdomys pumilo	*	MNHN 2005 688
Rhabdomys pumio	*	MNHN 2000 837
Rhynchomys sp.	Musser and Heaney [1]	FMNH 62289
Solomys sapientis	Misonne [2]	IRSNB 6368
Sommeromys macrorhinos	Musser and Durden [6]	

Spelaeomys florensis	Misonne [2]		
Stochomys longicaudatus	*		MNHN 1996 498
Stochomys longicaudatus	*		MNHN 1996 501
Sundamys mulleri	*		MNHN 1990 569
Tateomys macrocercus	Musser and Durden [6]		AMNH 225077
Thallomys paeduculus	*		MNHN 1977 24
Thammomys sp.	*		MNHN AC1967 362
Tokudaia sp.	Kaneko [15]		
Uromys caudimaculatus	Misonne [2]		IRSNB 4007
Uromys validus	Tate [4]		AM104507
Vandeluria oleracea	*		MNHN 1969 276
Vernaya fulva	Misonne [2]		AMNH 115467
Zelotomys hildegardeae	*		MNHN 2000 114
Zyzomys rackhami	Godthelp [16]		
Anthracomys lorenzi	Casanovas-Vilar et al. [17]	Fiume Santo	FS-779
Anthracomys majori	Casanovas-Vilar et al. [17]	Monte Bamboli	B5
Castillomys cf. crusafonti	García-Alix [18], García-Alix et al. [19]	Barranco de Blas	BLS-6 10
Castillomys cf. crusafonti	Minwer-Barakat et al. [20]	Tollo Chiclana 1B	TCH-1B 356
Castillomys crusafonti	van de Weerd [21]	Caravaca	1081
Castillomys gracilis	Adrover [22]	Aldehuela	ALD 116
Castillomys gracilis	Adrover [22]	Villaba Alta	VA1 185
Castillomys gracilis	Bachelet [23]	La Gloria 4	LG4 112
Castillomys gracilis	García-Alix [18]	Calicasas 3	CLC-3 30
Castillomys gracilis	Martín-Suárez [24]	Botardo-C	Bo-C 08
Castillomys gracilis	Martín-Suárez [24]	Botardo-C	Bo-C 09
Castromys inflatus	Martín-Suárez and Freudenthal [25]	Crevillente 31	RGM 413 759
Castromys littoralis	Freudenthal and Martín-Suárez [26]	Crevillente 17	RGM 413 092
Castromys littoralis	Freudenthal and Martín-Suárez [26]	Crevillente 17	RGM 413 009
Castromys littoralis	García-Alix [18], García-Alix et al. [19]	Jun 2B	JUN-2B 18
Castromys littoralis	García-Alix [18], García-Alix et al. [19]	Jun 2B	JUN-2B 19
Castromys littoralis	Martín-Suárez and Freudenthal [25]	Crevillente 22	CR22-24
Castromys littoralis	Martín-Suárez and Freudenthal [25]	Crevillente 17	RGM 413 103
Huerzelerimys minor	Adrover et al. [27]	La Gloria 4	LG4 15
Huerzelerimys minor	Mein et al. [28]	Ambérieu 2C	
Huerzelerimys minor	Mein et al. [28]	Ambérieu 2C	
Huerzelerimys minor	Mein et al. [28]	Cascante	
Huerzelerimys minor	Mein et al. [28]	Cortijo de Piedra 2	
Huerzelerimys minor	Mein et al. [28]	Cucalón	
Huerzelerimys turolensis	Adrover [22]	Aljezar B	AB 921
Huerzelerimys turolensis	Adrover [22]	Aljezar B	AB 932
Huerzelerimys turolensis	Adrover [22]	Aljezar B	AB 933
Huerzelerimys turolensis	Adrover [22]	Aljezar B	AB 969
Huerzelerimys turolensis	Adrover [22]	Aljezar B	AB 970
Huerzelerimys turolensis	Adrover [22]	Aljezar B	AB 974
Huerzelerimys turolensis	Adrover [22]	Aljezar B	AB 974
Huerzelerimys turolensis	Adrover [22]	Aljezar B	AB944
Huerzelerimys turolensis	Adrover [22]	Aljezar B	AB975

Huerzelerimys turolensis	Aguilar et al. [29]	Castelnou 1	CTN56
Huerzelerimys turolensis	Martín-Suárez and Freudenthal [30]	Crevillente 15	RGM 403 833
Huerzelerimys vireti	Adrover [22]	Los Aguanaces	LA 300
Huerzelerimys vireti	Adrover [22]	Los Aguanaces	LA 301
Huerzelerimys vireti	Adrover [22]	Los Aguanaces	LA 306
Huerzelerimys vireti	Adrover [22]	Vivero de Pinos	VP 312
Huerzelerimys vireti	Adrover [22]	Vivero de Pinos	VP 315
Huerzelerimys vireti	Alberdi et al. [31]	Puente Minero	PM-1424
Huerzelerimys vireti	Martín-Suárez and Freudenthal [30]	Crevillente 2	RGM 402 373
Huerzelerimys vireti	Martín-Suárez and Freudenthal [30]	Crevillente 2	RGM 402 384
Huerzelerimys vireti	Martín-Suárez and Freudenthal [30]	Crevillente 4B	RGM 404 046
Occitanomys adroveri	Adrover [22]	Aljezar B	AB 5
Occitanomys adroveri	Adrover [22]	Aljezar B	AB 61
Occitanomys adroveri	Adrover [22]	Aljezar B	AB 78
Occitanomys adroveri	Aguilar et al. [32]	Castelnou 3	CTN3 155
Occitanomys adroveri	Freudenthal and Martín-Suárez [26]	Crevillente 17	RGM 413 248
Occitanomys adroveri	García-Alix [18]	Otura-1	OTU-1 90
Occitanomys adroveri	García-Alix [18], García-Alix et al. [19]	Otura-1	OTU-1 44
Occitanomys adroveri	Martín-Suárez and Freudenthal [30]	Crevillente 15	RGM 403 670
Occitanomys adroveri	Martín-Suárez and Freudenthal [30]	Crevillente 15	RGM 403 679
Occitanomys adroveri	Martín-Suárez and Freudenthal [30]	Crevillente 17	RGM 413 345
Occitanomys adroveri	Martín-Suárez [24]	Botardo-C	Bo-C 16
Occitanomys alcalai	Adrover et al. [27]	La Gloria 4	LG4 26
Occitanomys alcalai	Adrover et al. [27]	La Gloria 4	LG4 31
Occitanomys alcalai	García-Alix [18], García-Alix et al. [19]	Purcal-4	PUR-4 39
Occitanomys alcalai	Minwer-Barakat et al. [33]	Rambla de Chimeneas 3	RCH-3 17
Occitanomys faillati	Aguilar et al. [29]	Castelnou 1	CTN1
Occitanomys sondaari	Adrover [22]	Los Aguanaces	LA 1
Occitanomys sondaari	Adrover [22]	Los Aguanaces	LA 22
Occitanomys sondaari	Adrover [22]	Los Aguanaces	LA 243
Occitanomys sondaari	Adrover [22]	Los Aguanaces	LA 245
Occitanomys sondaari	Adrover [22]	Los Aguanaces	LA 246
Occitanomys sondaari	Adrover [22]	Los Aguanaces	LA 50
Occitanomys sondaari	Adrover [22]	Vivero de Pinos	VP 10
Occitanomys sondaari	Adrover [22]	Vivero de Pinos	VP 262
Occitanomys sondaari	Adrover [22]	Vivero de Pinos	VP 274
Occitanomys sondaari	Adrover [22]	Vivero de Pinos	VP 275
Occitanomys sondaari	Adrover [22]	Vivero de Pinos	VP 30
Occitanomys sondaari	Adrover [22]	Vivero de Pinos	VP 43
Occitanomys sondaari	Alcalá [34]	Puente Minero	PM-1160
Occitanomys sondaari	Alcalá [34]	Puente Minero	PM-1173
Occitanomys sondaari	Alcalá [34]	Puente Minero	PM-1178
Occitanomys sondaari	Martín-Suárez and Freudenthal [30]	Crevillente 2	RGM 402 596
Occitanomys sondaari	Martín-Suárez and Freudenthal [30]	Crevillente 2	RGM 402 598

Occitanomys sondaari	Martín-Suárez and Freudenthal [30]	Crevillente 2	RGM 402 616
Occitanomys sondaari	Martín-Suárez and Freudenthal [30]	Crevillente 4B	RGM 404 459
Occitanomys sondaari	Martín-Suárez and Freudenthal [30]	Crevillente 4B	RGM 404 466
Paraethomis cf. meini	Ruiz Bustos et al. [35]	Gorafe A	G21
Paraethomys aff. abaigari	García-Alix [18], García-Alix et al. [19]	Purcal-13	PUR-13 41
Paraethomys meini	Adrover et al. [27]	La Gloria 4	LG4 54
Paraethomys meini	Adrover [22]	Aldehuela	ALD 65
Paraethomys meini	Adrover [22]	Arquillo 3	AR3 32
Paraethomys meini	Adrover [22]	Villalba Alta	VAL 280
Paraethomys meini	García-Alix [18]	Mina 4	MNA-4 61
Paraethomys meini	García-Alix [18], García-Alix et al. [19]	Purcal-3	PUR-3 25
Paraethomys meini	Martín-Suárez [24]	Botardo-C	Bo-C 23
Progonomys cathalai	Agustí [36]	Torrent de Febulines	
Progonomys cathalai	Mein et al. [28]	Ambérieu	
Progonomys cathalai	Mein et al. [28]	Bayraktepe	Bayraktepe II
Progonomys cathalai	Mein et al. [28]	Biodrak	
Progonomys cathalai	Mein et al. [28]	Masia del barbo 2B	
Progonomys cathalai	Mein et al. [28]	Montredon	
Progonomys cathalai	Mein et al. [28]	Soblay	
Progonomys cathalai	Wessels [37]	Altintas 1	
Progonomys cathalai	Wessels [37]	Altintas 1	
Progonomys cathalai	Wessels [37]	Altintas 1	
Progonomys cathalai	Wessels [37]	Altintas 1	
Progonomys cathalai	Wessels [37]	Altintas 1	
Progonomys cathalai	Wessels [37]	Altintas 1	
Progonomys cathalai	Wessels [37]	Altintas 1	
Progonomys cathalai	Wessels [37]	Altintas 2	
Progonomys cathalai	Wessels [37]	Altintas 2	
Progonomys cathalai	Wessels [37]	Kütahya	
Progonomys cathalai	Wessels [37]	Kütahya	
Progonomys cf. woelferi	Guerra-Merchan et al. [38]	Racor	Ra-4
Progonomys clauzoni	Lazzari et al. [39]	Lo Fournas 16M	FOU16M 758
Progonomys clauzoni	Lazzari et al. [39]	Lo Fournas 16M	FOU16M 759
Progonomys clauzoni	Lazzari et al. [39]	Lo Fournas 16M	FOU16M 760
Progonomys clauzoni	Lazzari et al. [39]	Lo Fournas 16M	FOU16M 761
Progonomys clauzoni	Lazzari et al. [39]	Lo Fournas 16M	FOU16M 762
Progonomys clauzoni	Lazzari et al. [39]	Lo Fournas 16M	FOU16M 763
Progonomys clauzoni	Lazzari et al. [39]	Lo Fournas 16M	FOU16M 764
Progonomys clauzoni	Lazzari et al. [39]	Lo Fournas 16M	FOU16M 769
Progonomys clauzoni	Lazzari et al. [39]	Lo Fournas 16M	FOU16M 772
Progonomys clauzoni	Lazzari et al. [39]	Lo Fournas 16M	FOU16M 778
Progonomys clauzoni	Lazzari et al. [39]	Lo Fournas 16M	FOU16M 780
Progonomys clauzoni	Lazzari et al. [39]	Lo Fournas 16M	FOU16M 790
Progonomys clauzoni	Lazzari et al. [39]	Lo Fournas 16M	FOU16M 795
Progonomys clauzoni	Lazzari et al. [39]	Lo Fournas 16M	FOU16M 820
Progonomys clauzoni	Lazzari et al. [39]	Lo Fournas 16M	FOU16M 846
Progonomys clauzoni	Lazzari et al. [39]	Lo Fournas 6a	FOU6a 103
Progonomys clauzoni	Lazzari et al. [39]	Lo Fournas 6a	FOU6a 114
Progonomys clauzoni	Lazzari et al. [39]	Lo Fournas 6a	FOU6a 155
Progonomys clauzoni	Lazzari et al. [39]	Lo Fournas 6a	FOU6a 156
Progonomys clauzoni	Lazzari et al. [39]	Lo Fournas 6a	FOU6a 157

Progonomys clauzoni	Lazzari et al. [39]	Lo Fournas 6a	FOU6a 158
Progonomys clauzoni	Lazzari et al. [39]	Lo Fournas 6a	FOU6a 159
Progonomys clauzoni	Lazzari et al. [39]	Lo Fournas 6a	FOU6a 160
Progonomys clauzoni	Lazzari et al. [39]	Lo Fournas 6b	FOU6b 16
Progonomys clauzoni	Lazzari et al. [39]	Lo Fournas 6b	FOU6b 19
Progonomys clauzoni	Lazzari et al. [39]	Lo Fournas 6b	FOU6b 28
Progonomys clauzoni	Lazzari et al. [39]	Lo Fournas 6b	FOU6b 44
Progonomys clauzoni	Lazzari et al. [39]	Lo Fournas 6b	FOU6b 45
Progonomys clauzoni	Lazzari et al. [39]	Lo Fournas 6b	FOU6b 46
Progonomys clauzoni	Lazzari et al. [39]	Lo Fournas 6b	FOU6b 8
Progonomys clauzoni	Lazzari et al. [39]	Lo Fournas 6c	FOU6c 1081
Progonomys clauzoni	Lazzari et al. [39]	Lo Fournas 6c	FOU6c 1082
Progonomys clauzoni	Lazzari et al. [39]	Lo Fournas 6c	FOU6c 1083
Progonomys clauzoni	Lazzari et al. [39]	Lo Fournas 6c	FOU6c 160
Progonomys clauzoni	Lazzari et al. [39]	Lo Fournas 6c	FOU6c 196
Progonomys clauzoni	Lazzari et al. [39]	Lo Fournas 6c	FOU6c 206
Progonomys clauzoni	Lazzari et al. [39]	Lo Fournas 6c	FOU6c 207
Progonomys clauzoni	Lazzari et al. [39]	Lo Fournas 6c	FOU6c 210
Progonomys hispanicus	Antunes et al. [40]	Asseiceira	
Progonomys hispanicus	Sesé [41]	Belmonte	BE-3
Progonomys hispanicus	Sesé [41]	Belmonte	BE-4
Progonomys woelferi	Mein et al. [28]	Torrent de Febulines	
Progonomys woelferi	Mein et al. [28]	Kastellios	
Progonomys woelferi	Mein et al. [28]	Kohfidisch	
Progonomys woelferi	Mein et al. [28]	YGSP	YGSP 182A
Rhagapodemus primaevus	Aguilar et al. [32]	Castenou 3	CTN 3 208
Rhagapodemus sp.	Adrover et al. [27]	La Gloria 4	LG4 121
Stephanomys cf. cordii	Ruiz Bustos et al. [35]	Gorafe A	G1
Stephanomys dubari	Aguilar et al. [32]	Castenou 3	CTN 3 79
Stephanomys dubari	Aguilar et al. [32]	Castenou 3	CTN 3 83
Stephanomys dubari	Aguilar et al. [32]	Castenou 3	CTN 3 85
Stephanomys dubari	Aguilar et al. [32]	Castenou 3	CTN 3 94
Stephanomys dubari	García-Alix [18]	Dehesa-16	DHS-16 129
Stephanomys dubari	García-Alix [18]	Purcal-13	PUR-13 112
Stephanomys ramblensis	Adrover et al. [27]	La Gloria 5	LG5 103
Stephanomys ramblensis	García-Alix [18]	Purcal-24	PUR-24 81
Stephanomys ramblensis	Sanz et al. [42]	Canteras de Iberia	
Stephanomys ramblensis	Sanz et al. [42]	Canteras de Iberia	
Stephanomys stadii	Mein and Michaux [43]	Cucuron	FSL 65626
Stephanomys stadii	Mein and Michaux [43]	Cucuron	FSL 65627
Stephanomys stadii	Mein and Michaux [43]	Cucuron	FSL 65628

References:

1. Musser GG, Heaney LR (1992) Philippine Rodents: Definitions of Tarsomys and Limnomys plus a preliminary assessment of phylogenetic patterns among native philippine murines (Murinae, Muridae). Bulletin of the American Museum of Natural History 211: 1-144.
2. Misonne X (1969) African and Indo-Australian Muridae: Musee Royal de L'Afrique Centrale - Tervuren, Belguique Annales Serie Nº 8. 1-185 p.

3. Durden L, Musser G (1991) A new species of sucking louse (Insecta, Anoplura) from a Montane forest rat in central sulawesi and a preliminary interpretation of the sucking louse fauna of sulawesi. american Museum Novitates 3008: 1-10.
4. Tate GHH (1936) Some Muridae of the Indo-Australian Region. Contained in Bulletin of the American Museum of Natural History, Volume 72, Issue 6 pages 501-728. Bulletin of the American Museum of Natural History 72: 501-728.
5. Musser GG, Lunde DP (2009) Systematic Reviews of New Guinea Coccymys and "Melomys" Albidens (Muridae, Murinae) with Descriptions of New Taxa. Bulletin of the American Museum of Natural History 329: 1-139.
6. Musser GG, Durden LA (2002) Sulawesi rodents: Description of a new genus and species of Murinae (Muridae, Rodentia) and its parasitic new species of sucking louse (Insecta, Anoplura) (American Museum novitates). American Museum Novitates 3368: 1-50.
7. Musser GG (1990) Sulawesi Rodents: Species Traits and ChromosomesofHaeromys minahassaeand Echiothrixleucura(Muridae:Murinae). American Museum Novitates 2989: 1-20.
8. Musser GG (1987) The occurrence of Hadromys (Rodentia, Muridae) in early Pleistocene Siwalik strata in northern Pakistan and its bearing on biogeographic affinities between Indian and northeastern African murine faunas. American Museum Novitates: 1-36.
9. Helgen KM (2005) The amphibious murines of New Guinea (Rodentia, Muridae): the generic status of Baiyankamys and description of a new species of Hydromys. Zootaxa 913: 1-20.
10. Cooper NK, Adams M, Anthony C (2003) Morphological and genetic variation in Leggadina (Thomas, 1910) with special reference to Western Australian populations. RECORDS-WESTERN ….
11. Musser GG (1982) Results of the Archbold Expeditions. No. 108. The Definition of Apomys, a Native Rat of the Philippine Islands. American Museum Novitates 2746: 1-44.
12. Musser GG (1981) RESULTS OF THE ARCHBOLD EXPEDITIONS. NO. 105. NOTES ON SYSTEMATICS OF INDO-MALAYAN MURID RODENTS, AND DESCRIPTIONS OF NEW GENERA AND SPECIES FROM CEYLON, SULAWESI, AND THE PHILIPPINES. Bulletin of the American Museum of Natural History 168: 225-334.
13. Kitchener DJ, How RA, Maharadatunkamsi (1991) Paulamys sp. cf. P. naso (Musser, 1981)(Rodentia: Muridae) from Flores Island, Nusa Tenggara, Indonesia—description from a modern specimen and a consideration of its phylogenetic affinities. Records of the West Australian Museum 15: 171-189.
14. Helgen KM, Helgen LE (2009) Chapter 8. Biodiversity and Biogeography of the Moss-mice of New Guinea: A Taxonomic Revision of Pseudohydromys (Muridae: Murinae). Bulletin of the American Museum of Natural History 331: 230-313.
15. Kaneko Y (2001) Morphological discrimination of the Ryukyu spiny rat (genus Tokudaia) between the islands of Okinawa and Amami Oshima, in the Ryukyu Islands, southern Japan. Mammal Study 26: 17-33.
16. Godthelp H (1997) ZYZOMYS RACKHAMI SP. NOV. (RODENTIA, MURIDAE) A ROCKRAT FROM PLIOCENE RACKHAM'S ROOST SITE, RIVERSLEIGH, NORTHWESTERN Queensland. Memoirs of the Queensland Museum 41: 329-333.
17. Casanovas-Vilar I, Van Dam jA, Moyà-Solà S, Rook L (2011) Late Miocene insular mice from the Tusco-Sardinian palaeobioprovince provide new insights on the

palaeoecology of the Oreopithecus faunas. Journal of Human Evolution 61: 42-49.
18. García-Alix A (2006) Bioestratigrafía de los depósitos continentales de la transición Mio-Plioceno de la cuenca de Granada. Granada Universidad de Granada. 386 p.
19. García-Alix A, Minwer-Barakat R, Martín JM, Martín-Suárez E, Freudenthal M (2008) Biostratigraphy and sedimentary evolution of Late Miocene and Pliocene continental deposits of the Granada Basin (southern Spain). Lethaia 41: 431-446.
20. Minwer-Barakat R, García-Alix A, Martín-Suárez E, Freudenthal M (2005) Muridae (rodentia) from the Pliocene of Tollo de Chiclana (Granada, southeastern Spain). Journal of Vertebrate Paleontology 25: 426-441.
21. van de Weerd A (1976): Utrecht Micropal. Bull. 216 p.
22. Adrover R (1986) Nuevas faunas de roedores en el Mio-Plioceno continental de la region de Teruel (España). Interés bioestratigráfico y paleoecológico. Teruel: Instituto de Estudios Turolenses. Diputación Provovincial de Teruel. 423 p.
23. Bachelet B (1990) Muridae et Arvicolidae (Rodentia, Mammalia) du Pliocène du Sud dela France: Systématique, Évolution, Biochronologie. [Doctoral Tesis]. Montpellier: Univ. Montpellier.
24. Martín-Suárez E (1988) Sucesiones de micromamíferos en la Depresión Guadix-Baza (Granada, España). Granada: Universidad de Granada. 241 p.
25. Martín-Suárez E, Freudenthal M (1994) Castromys, a new genus of Muridae (Rodentia) from the Late Miocene of Spain. Scripta Geologica 106: 11-34.
26. Freudenthal M, Martín-Suárez E (1999) Family Muridae. In: Rössner G, Heissig K, editors. The Miocene land mammals of Europe. München: Verlang Dr. Friedrich Pfeil. pp. 401-409.
27. Adrover R, Mein P, Moissenet E (1993) Roedores de la transición Mio-Plioceno de la región de Teruel. Paleontologia i Evolucio 26-27: 47-84.
28. Mein P, Martín-Suárez E, Agustí J (1993) Progonomys Schaub, 1938 and Huerzelerimys gen. nov.(Rodentia); their evolution in Western Europe. Scripta Geologica 103: 41-64.
29. Aguilar JP, CALVET M, Michaux J (1995) Les Rongeurs du gisement karstique Miocène supérieur de Castelnou 1 (Pyrénées-Orientales, France). Geobios 28: 501-510.
30. Martín-Suárez E, Freudenthal M (1993) Muridae (Rodentia) from the Lower Turolian of Crevillente (Alicante, Spain). Scripta Geologica 103.
31. Alberdi MT, Alcalá L, Azanza B, Cerdeño E, Mazo AV, et al. (1989) Consideraciones biostratigráficas sobre la fauna de Vertebrados fósiles de la cuenca de Guadix-Baza (Granada, España).
32. Aguilar J-P, Michaux J, Bachelet B, Calvet M, J.-P. F (1991) Les nouvelles faunes de rongeurs proches de la limite mio-pliocene en Roussillon. Implications biostratigraphiques et biogéographiques. Palaeovertebrata 20: 147-174.
33. Minwer-Barakat R, García-Alix A, Martín-Suárez E, Freudenthal M (2009) Late Turolian micromammals from Rambla de Chimeneas-3: considerations on the oldest continental faunas from the Guadix Basin (Southern Spain). Neues Jahrbuch für Geologie und Paläontologie - Abhandlungen 251: 95-108.
34. Alcalá L, Sesé C, Herraez E, Adrover R (1991) Mamíferos del Turoliense inferior de Puente Minero (Teruel, España). Bol R Soc Esp Hist Nat (Sec Geol) 86: 205-251.
35. Ruiz Bustos A, Sesé C, Dabrio CJ (1984) Geología y fauna de micromamíferos del nuevo yacimiento del Plioceno inferior de Gorafe-A (Depresión de Guadix-Baza, Granada). Estudios Geológicos.

36. Agustí J (1981) Roedores miomorphos del Neogeno de Cataluña. Barcelona: Universidad de Barcelona. 293 p.
37. Wessels W (1955) Miocene rodent evolution and migration Muroidea from Pakistan, Turkey and Northern Africa: Utrecht University. 289 p.
38. Guerra-Merchan A, Ramallo D, Ruiz Bustos A (2001) New data on the upper miocene micromammals of the Betic cordillera and their interest form marine-continental correlations. Geobios 34: 85-90.
39. Lazzari V, Aguilar J-P, Michaux J (2010) Intraspecific variation and micro-macroevolution connection: illustration with the late Miocene genus Progonomys (Rodentia, Muridae). Paleobiology 36: 641-657.
40. Antunes MT, Soulie-Marsche I, Mein P, Pais J (1992) Le gisement de Asseiceira, Portugal (Miocene superieur). Donnees complementaires sur Freiria de Rio Maior. Universidade Nova de Lisboa Ciencias da Terra 11.
41. Sesé C (2003) Paleontologia y bioestratigrafia del mioceno continental de la Cuenca de Calatayud (Zaragoza): nuevos yacimientos de micromamiferos. Estudios Geológicos 59: 249-264.
42. Sanz E, Sesé C, Calvo JP (1992) Primer hallazgo de micromamíferos de edad Turoliense en la Cuenca de Madrid. Estudios Geológicos 48: 171-178.
43. Mein P, Michaux J (1979) Une faune de petits mammifères d'âge Turolien moyen (Miocène supérieur) à Cucuron (Vaucluse) données nouvelles sur le genre Stephanomys (Rod.) et conséquences stratigraphiques. Geobios, paléontologie, stratigraphie et paléoécologie 12: 481-485.
