# Supplementary material for: Dietary Ecology of Murinae (Muridae, Rodentia): A Geometric Morphometric Approach
Source: PLoS One. 2013 Nov 13;8(11):e79080. doi: 10.1371/journal.pone.0079080 (PMC3827291; doi:10.1371/journal.pone.0079080)
Supplement: Appendix S3 — Estimation of the information content in each harmonic based on its amplitude. The amplitudes were cumulated over the total range of harmonics and the information brought by each harmonic was estimated as the percentage of the sum of all harmonic amplitudes. In our case each of the nine first harmonics increased the amount of shape information up to 97% of the total information, meanwhile the subsequent harmonics provided almost no further relevant shape information (Fig. S1). (PDF) [file pone.0079080.s003.pdf]

Appendix S3. Following the consideration in Crampton [1] , the information content in each harmonic was estimated based on the amplitude of the harmonics. The amplitudes were cumulated over the total range of harmonics and the information brought by each harmonic was estimated as the percentage of the sum of all harmonic amplitudes [2]. In our case each of the nine first harmonics increased the amount of shape information up to 97% of the total information, meanwhile the subsequent harmonics provided almost no further relevant shape information (Fig. S1).

Therefore, the first nine harmonics appear to provide a good compromise between information content and the number of variables to be considered.

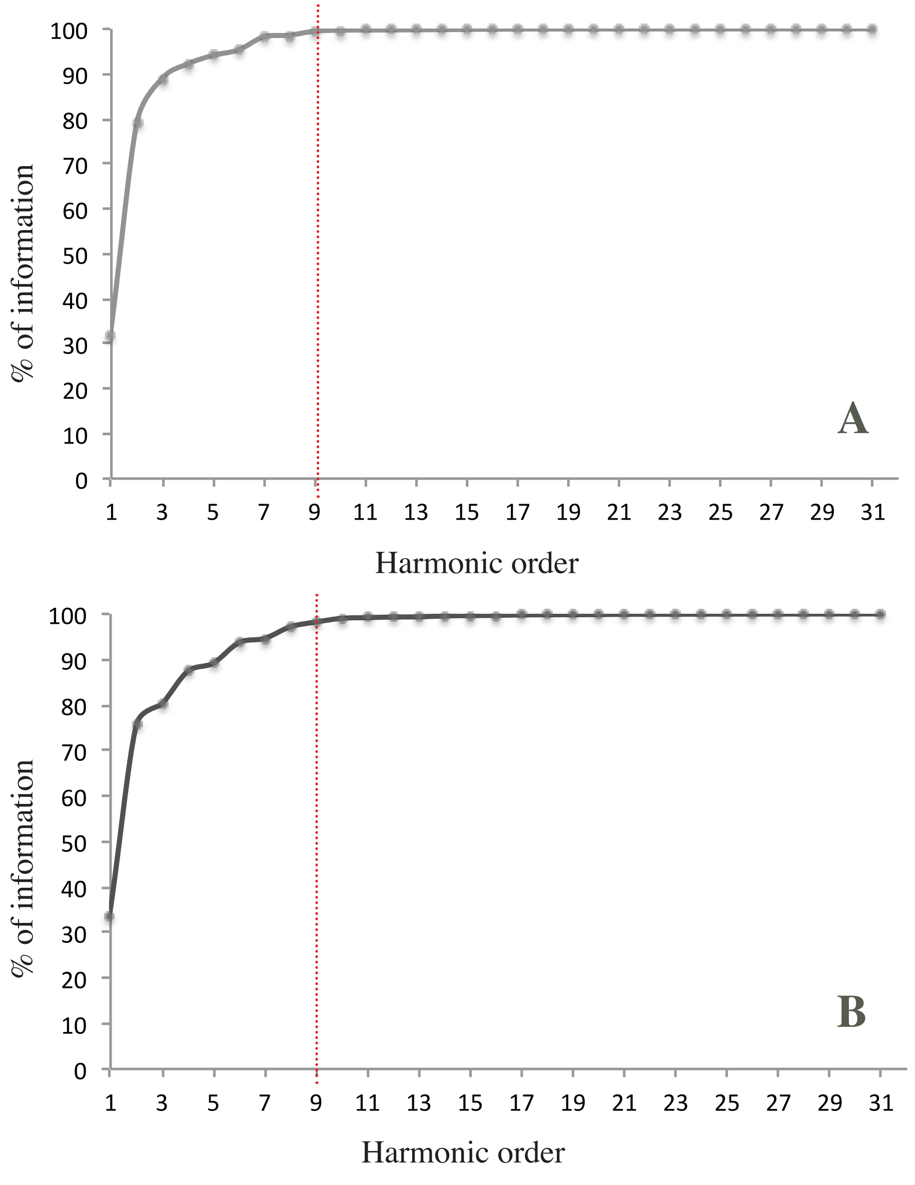

Figure S1. Cumulative power as a function of the harmonic order for the Elliptic Fourier transform of (A) one draw (*Apodemus atavus* 4150) and (B) for one picture (*Coryphomys buehleri* AMF68751). The cumulative power correspond to the contribution of each harmonic (%) to the total information.

Refernces

1. Crampton JS (1995) Elliptic Fourier shape analysis of the fossil bivalves: some practical considerations. Lethaia 28: 179-186.
2. Helvaci Z, Renaud S, Ledevin R, Adriaens D, Michaux J, et al. (2012) Morphometric and genetic structure of the edible dormouse (*Glis glis*): a consequence of forest fragmentation in Turkey. Biological Journal of the Linnean Society 107: 611-623.
