# Supplementary material for: Dietary Ecology of Murinae (Muridae, Rodentia): A Geometric Morphometric Approach
Source: PLoS One. 2013 Nov 13;8(11):e79080. doi: 10.1371/journal.pone.0079080 (PMC3827291; doi:10.1371/journal.pone.0079080)
Supplement: Appendix S4 — Fourier Components and size. Values estimated for each extant and extinct genus considered in this works after EFA. (PDF) [file pone.0079080.s004.pdf]

Appendix S4. Table with the calculated FC and estimated size for each extant and extinct genus considered in this works after EFA

Genus		Size	A1	B1	C1	D1	A2	B2	C2
Abditomys	Extant	1.403	1.000	0.000	0.000	-0.732	0.008	-0.037	0.039
Aethomys	Extant	1.233	1.000	0.000	0.000	-0.802	0.023	-0.015	0.029
Anisomys	Extant	2.922	1.000	0.000	0.000	-0.736	0.016	-0.011	0.032
Apodemus	Extant	0.880	1.000	0.000	0.000	-0.739	0.022	-0.018	0.035
Apomys	Extant	1.108	1.000	0.000	0.000	-0.566	0.006	-0.012	0.031
Archboldomys	Extant	1.144	1.000	0.000	0.000	-0.729	-0.015	-0.043	0.032
Arvicanthis	Extant	1.305	1.000	0.000	0.000	-0.810	0.016	-0.026	0.025
Bandicota	Extant	1.959	1.000	0.000	0.000	-0.774	0.016	-0.033	0.056
Bunomys	Extant	1.537	1.000	0.000	0.000	-0.731	0.009	-0.032	0.046
Chiropodomys	Extant	0.965	1.000	0.000	0.000	-0.737	0.010	-0.017	0.029
Chrotomys	Extant	1.464	1.000	0.000	0.000	-0.591	-0.001	-0.026	0.052
Coccymys	Extant	0.995	1.000	0.000	0.000	-0.718	0.018	-0.021	0.020
Colomys	Extant	1.557	1.000	0.000	0.000	-0.691	0.009	-0.020	0.046
Crateromys	Extant	3.113	1.000	0.000	0.000	-0.717	0.020	-0.022	0.032
Crunomys	Extant	1.067	1.000	0.000	0.000	-0.683	0.012	-0.036	0.036
Dasymys	Extant	1.402	1.000	0.000	0.000	-0.835	0.005	-0.033	0.032
Echiothrix	Extant	1.559	1.000	0.000	0.000	-0.726	0.014	-0.021	0.026
Eropeplus	Extant	1.905	1.000	0.000	0.000	-0.758	0.016	-0.010	0.075
Golunda	Extant	1.248	1.000	0.000	0.000	-0.881	-0.015	-0.031	0.029
Grammomys	Extant	0.977	1.000	0.000	0.000	-0.741	0.013	-0.018	0.032
Hadromys	Extant	1.420	1.000	0.000	0.000	-0.758	-0.007	-0.034	0.033
Haeromys	Extant	0.717	1.000	0.000	0.000	-0.719	0.010	-0.023	0.043
Hapalomys	Extant	1.504	1.000	0.000	0.000	-0.724	0.004	0.002	0.006
Heimyscus	Extant	0.884	1.000	0.000	0.000	-0.749	0.021	-0.020	0.066
Hybomys	Extant	1.154	1.000	0.000	0.000	-0.775	0.017	-0.017	0.023
Hydromys	Extant	1.660	1.000	0.000	0.000	-0.557	0.016	-0.028	0.043
Hylomyscus	Extant	0.827	1.000	0.000	0.000	-0.670	0.017	-0.016	0.043
Hyomys	Extant	1.020	1.000	0.000	0.000	-0.752	0.007	-0.023	0.038
Kadarsanomys	Extant	1.595	1.000	0.000	0.000	-0.751	0.001	-0.019	0.014
Leggadina	Extant	1.033	1.000	0.000	0.000	-0.681	0.010	-0.019	0.027
Lemniscomys	Extant	1.143	1.000	0.000	0.000	-0.766	0.018	-0.028	0.041
Lenomys	Extant	1.992	1.000	0.000	0.000	-0.750	0.017	-0.014	0.040
Lenothrix	Extant	0.000	1.000	0.000	0.000	-0.734	-0.001	-0.017	0.064
Leopoldomys	Extant	2.161	1.000	0.000	0.000	-0.677	0.004	-0.029	0.054
Leporillus	Extant	1.539	1.000	0.000	0.000	-0.752	0.008	-0.028	0.036
Leptomys	Extant	1.549	1.000	0.000	0.000	-0.579	0.004	-0.016	0.060
Lorentzimys	Extant	1.174	1.000	0.000	0.000	-0.747	0.013	-0.040	0.055
Malacomys	Extant	1.527	1.000	0.000	0.000	-0.702	0.010	-0.023	0.047
Mallomys	Extant	2.802	1.000	0.000	0.000	-0.765	0.001	-0.024	0.033
Margaretamys	Extant	1.219	1.000	0.000	0.000	-0.651	0.002	-0.027	0.044
Mastacomys	Extant	1.773	1.000	0.000	0.000	-0.874	-0.028	-0.053	0.043
Mastomys	Extant	1.126	1.000	0.000	0.000	-0.698	0.012	-0.029	0.074
Maxomys	Extant	1.466	1.000	0.000	0.000	-0.702	0.017	-0.024	0.036

Genus		Size	A1	B1	C1	D1	A2	B2	C2
Melasmothrix	Extant	1.319	1.000	0.000	0.000	-0.764	0.035	0.000	0.054
Melomys	Extant	1.623	1.000	0.000	0.000	-0.686	0.015	-0.009	0.017
Micromys	Extant	0.607	1.000	0.000	0.000	-0.714	0.006	-0.009	0.008
Millardia	Extant	1.172	1.000	0.000	0.000	-0.788	0.017	-0.030	0.034
Mus	Extant	0.875	1.000	0.000	0.000	-0.664	0.015	-0.023	0.046
Nivivemter	Extant	1.281	1.000	0.000	0.000	-0.638	0.005	-0.027	0.041
Notomys	Extant	1.132	1.000	0.000	0.000	-0.768	0.010	-0.036	0.054
Oenomys	Extant	1.413	1.000	0.000	0.000	-0.793	0.006	-0.022	0.019
Papagonomys	Extant	4.339	1.000	0.000	0.000	-0.759	-0.001	-0.033	0.027
Parahydromys	Extant	2.469	1.000	0.000	0.000	-0.621	-0.003	0.014	-0.072
Paulamys	Extant	1.585	1.000	0.000	0.000	-0.667	0.013	-0.022	0.036
Pelomys	Extant	1.357	1.000	0.000	0.000	-0.862	0.007	-0.033	0.031
Phloeomys	Extant	3.494	1.000	0.000	0.000	-0.754	0.032	0.001	0.010
Pitecheir	Extant	1.908	1.000	0.000	0.000	-0.632	0.012	-0.019	0.044
Pogonomys	Extant	1.206	1.000	0.000	0.000	-0.801	0.004	-0.013	0.032
Praomys	Extant	1.051	1.000	0.000	0.000	-0.679	0.012	-0.023	0.039
Pseudohydromys	Extant	0.756	1.000	0.000	0.000	-0.670	0.047	-0.020	0.032
Pseudomys	Extant	1.069	1.000	0.000	0.000	-0.826	0.007	-0.031	0.026
Rattus	Extant	1.440	1.000	0.000	0.000	-0.677	0.010	-0.027	0.039
Rhabdomys	Extant	0.965	1.000	0.000	0.000	-0.755	0.025	-0.012	0.010
Rhynchomys	Extant	0.634	1.000	0.000	0.000	-0.609	0.014	-0.014	0.012
Solomys	Extant	2.811	1.000	0.000	0.000	-0.710	0.006	-0.024	0.026
Sommeromys	Extant	0.998	1.000	0.000	0.000	-0.716	0.001	-0.012	0.027
Spelaeomys	Extant	4.260	1.000	0.000	0.000	-0.750	0.005	0.001	0.005
Stochomys	Extant	1.423	1.000	0.000	0.000	-0.766	0.015	-0.018	0.034
Sundamys	Extant	2.332	1.000	0.000	0.000	-0.620	0.009	-0.018	0.056
Tateomys	Extant	1.168	1.000	0.000	0.000	-0.789	0.002	-0.049	0.003
Thallomys	Extant	1.030	1.000	0.000	0.000	-0.762	0.026	-0.022	0.030
Thammomys	Extant	1.187	1.000	0.000	0.000	-0.760	0.008	-0.019	0.024
Tokudaia	Extant	0.927	1.000	0.000	0.000	-0.685	0.007	-0.027	0.026
Uromys	Extant	2.381	1.000	0.000	0.000	-0.619	0.005	-0.020	0.049
Vandeleuria	Extant	0.715	1.000	0.000	0.000	-0.662	0.016	-0.018	0.003
Vernaya	Extant	2.221	1.000	0.000	0.000	-0.654	0.006	-0.008	0.039
Zelotomys	Extant	1.375	1.000	0.000	0.000	-0.759	0.011	-0.039	0.048
Zyzomys	Extant	1.338	1.000	0.000	0.000	-0.665	0.027	-0.013	0.016
Anthracomys	Extinct	1.491	1.000	0.000	0.000	-0.679	0.025	-0.039	0.037
Castillomys	Extinct	0.712	1.000	0.000	0.000	-0.744	0.024	-0.020	0.041
Castromys	Extinct	1.178	1.000	0.000	0.000	-0.757	0.016	-0.030	0.039
Huerzelerimys	Extinct	1.193	1.000	0.000	0.000	-0.732	0.014	-0.028	0.047
Occitanomys	Extinct	1.035	1.000	0.000	0.000	-0.773	0.025	-0.028	0.047
Paraethomys	Extinct	1.063	1.000	0.000	0.000	-0.730	0.012	-0.031	0.051
Progonomys	Extinct	0.890	1.000	0.000	0.000	-0.742	0.015	-0.032	0.052
Rhagapodemus	Extinct	0.961	1.000	0.000	0.000	-0.678	0.012	-0.017	0.035
Stephanomys	Extinct	1.048	1.000	0.000	0.000	-0.766	0.017	-0.024	0.038

Appendix D.

Genus	D2	A3	B3	C3	D3	A4	B4	C4	D4
Abditomys	0.009	0.061	0.005	-0.017	-0.015	0.006	-0.019	-0.012	-0.002
Aethomys	0.036	0.037	0.000	-0.014	-0.023	0.021	-0.011	-0.010	-0.003
Anisomys	0.022	0.033	0.005	-0.003	-0.064	0.007	-0.012	-0.009	-0.014
Apodemus	0.028	0.039	-0.007	-0.004	-0.047	0.021	-0.010	-0.011	-0.002
Apomys	0.015	0.069	0.002	-0.013	-0.059	0.008	-0.010	-0.004	-0.001
Archboldomys	-0.005	0.040	-0.009	-0.004	-0.067	-0.004	-0.037	0.020	0.001
Arvicanthis	0.033	0.041	-0.006	-0.008	-0.021	0.011	-0.015	-0.011	-0.009
Bandicota	0.025	0.032	-0.002	0.003	-0.042	0.010	-0.027	-0.017	-0.009
Bunomys	0.024	0.049	-0.009	-0.001	-0.041	0.010	-0.020	-0.009	-0.004
Chiropodomys	0.024	0.043	0.003	-0.004	-0.046	0.011	-0.008	-0.014	-0.009
Chrotomys	-0.002	0.064	-0.001	-0.005	-0.071	0.001	-0.020	-0.003	0.001
Coccymys	0.025	0.045	0.000	-0.001	-0.050	0.017	-0.015	0.007	-0.004
Colomys	0.012	0.047	-0.002	-0.005	-0.059	0.012	-0.013	-0.018	0.009
Crateromys	0.050	0.047	-0.014	0.005	-0.049	0.026	-0.015	-0.011	-0.010
Crunomys	0.022	0.056	-0.001	-0.007	-0.056	0.012	-0.020	0.000	-0.005
Dasymys	0.017	0.034	-0.009	0.007	-0.018	0.000	-0.022	-0.013	-0.014
Echiothrix	0.025	0.045	0.001	-0.006	-0.048	0.010	-0.008	-0.012	-0.009
Eropeplus	0.016	0.061	-0.003	-0.024	-0.040	0.013	-0.020	-0.020	0.006
Golunda	-0.008	0.015	-0.011	0.005	-0.011	-0.017	-0.020	-0.008	-0.007
Grammomys	0.021	0.043	-0.001	-0.007	-0.045	0.012	-0.012	-0.008	-0.002
Hadromys	0.002	0.054	-0.010	0.003	-0.013	-0.002	-0.022	-0.010	-0.003
Haeromys	0.010	0.048	0.009	-0.020	-0.046	0.009	-0.017	-0.011	-0.008
Hapalomys	-0.002	0.019	-0.002	-0.002	-0.088	0.005	0.003	-0.004	0.007
Heimyscus	0.017	0.039	-0.001	-0.024	-0.045	0.015	-0.025	-0.015	-0.001
Hybomys	0.042	0.044	0.001	-0.008	-0.033	0.021	-0.012	-0.012	-0.011
Hydromys	0.007	0.069	0.005	-0.005	-0.060	0.005	-0.017	-0.007	-0.002
Hylomyscus	0.023	0.054	-0.001	-0.012	-0.045	0.022	-0.018	-0.005	0.007
Hyomys	0.009	0.040	-0.003	-0.012	-0.044	0.004	-0.019	-0.009	-0.002
Kadarsanomys	0.019	0.059	0.011	-0.024	-0.030	0.009	0.005	-0.020	0.001
Leggadina	0.029	0.055	0.003	-0.025	-0.048	0.014	-0.011	-0.016	-0.008
Lemniscomys	0.031	0.041	-0.003	-0.009	-0.034	0.015	-0.019	-0.011	-0.005
Lenomys	0.029	0.044	-0.006	-0.001	-0.048	0.015	-0.009	-0.018	-0.005
Lenothrix	0.000	0.039	-0.001	-0.011	-0.062	0.012	-0.014	-0.028	0.012
Leopoldomys	0.008	0.054	0.003	-0.017	-0.044	0.007	-0.018	-0.018	-0.002
Leporillus	0.019	0.024	-0.002	-0.008	-0.065	0.007	-0.024	-0.009	-0.004
Leptomys	-0.009	0.066	0.005	-0.023	-0.075	0.003	-0.018	-0.003	0.005
Lorentzimys	0.023	0.041	0.006	-0.008	-0.040	0.012	-0.026	-0.018	-0.010
Malacomys	0.020	0.053	-0.005	-0.006	-0.045	0.013	-0.017	-0.016	0.003
Mallomys	0.017	0.052	0.001	-0.006	-0.050	0.016	-0.015	-0.016	-0.008
Margaretamys	-0.001	0.060	0.000	-0.013	-0.051	0.004	-0.019	-0.010	0.006
Mastacomys	-0.022	0.016	-0.007	-0.009	-0.025	-0.010	-0.038	0.003	0.010
Mastomys	0.023	0.052	0.001	-0.016	-0.042	0.013	-0.030	-0.019	-0.001
Maxomys	0.026	0.048	0.002	-0.016	-0.052	0.018	-0.018	-0.007	0.001

Genus	D2	A3	B3	C3	D3	A4	B4	C4	D4
Melasmothrix	0.031	0.054	0.007	-0.019	-0.048	0.029	-0.024	-0.007	0.010
Melomys	0.008	0.042	0.002	-0.007	-0.061	0.015	-0.008	-0.003	0.001
Micromys	0.028	0.058	-0.002	0.002	-0.041	0.010	-0.001	-0.012	-0.010
Millardia	0.037	0.046	-0.008	-0.008	-0.018	0.020	-0.024	-0.014	-0.008
Mus	0.040	0.064	-0.002	-0.030	-0.042	0.018	-0.010	-0.029	-0.001
Nivivemter	0.014	0.061	-0.002	-0.006	-0.051	0.008	-0.017	-0.008	0.002
Notomys	0.013	0.042	0.003	-0.012	-0.026	0.008	-0.026	-0.018	-0.002
Oenomys	0.014	0.035	0.002	-0.023	-0.026	0.021	-0.016	-0.002	0.013
Papagonomys	0.017	0.053	0.001	-0.003	-0.033	0.009	-0.012	-0.007	-0.004
Parahydromys	0.001	0.059	-0.006	0.000	-0.070	0.000	0.018	0.007	-0.006
Paulamys	0.027	0.071	-0.006	0.012	-0.011	0.006	-0.021	-0.008	-0.006
Pelomys	0.019	0.017	-0.010	0.000	-0.029	0.014	-0.029	-0.002	-0.001
Phloeomys	0.048	0.034	0.003	0.004	-0.055	0.022	-0.007	0.001	-0.019
Pitecheir	0.035	0.062	-0.006	-0.023	-0.053	0.028	-0.019	-0.022	0.010
Pogonomys	0.001	0.033	0.002	-0.015	-0.029	0.010	-0.017	-0.001	0.011
Praomys	0.035	0.055	-0.009	-0.006	-0.039	0.019	-0.018	-0.012	0.004
Pseudohydromys	0.046	0.033	0.009	-0.025	-0.076	0.034	-0.015	0.001	0.003
Pseudomys	0.007	0.028	0.002	-0.002	-0.033	0.003	-0.021	-0.008	-0.002
Rattus	0.030	0.059	-0.007	-0.003	-0.039	0.013	-0.020	-0.008	-0.003
Rhabdomys	0.040	0.043	0.005	-0.012	-0.035	0.022	-0.007	-0.002	-0.002
Rhynchomys	0.049	0.071	0.011	-0.023	-0.042	0.008	-0.008	0.000	-0.030
Solomys	0.011	0.052	-0.004	0.002	-0.038	0.007	-0.009	-0.018	-0.001
Sommeromys	-0.001	0.051	-0.004	0.003	-0.039	-0.001	-0.017	0.008	0.002
Spelaeomys	0.010	0.037	-0.007	0.011	-0.058	-0.007	0.005	-0.003	-0.018
Stochomys	0.022	0.039	0.001	-0.003	-0.040	0.006	-0.014	-0.009	-0.016
Sundamys	0.014	0.063	-0.004	-0.009	-0.052	0.009	-0.015	-0.013	-0.001
Tateomys	0.031	0.058	-0.004	-0.006	-0.021	0.019	-0.013	0.000	0.008
Thallomys	0.043	0.040	0.003	-0.013	-0.036	0.023	-0.017	-0.008	-0.005
Thammomys	0.010	0.035	-0.003	-0.008	-0.047	0.013	-0.017	0.002	0.004
Tokudaia	0.010	0.051	0.003	0.006	-0.078	-0.004	-0.007	-0.013	-0.025
Uromys	0.003	0.061	-0.002	-0.002	-0.055	0.007	-0.014	-0.007	0.003
Vandeluria	0.038	0.056	-0.008	0.013	-0.061	0.023	-0.005	-0.012	0.001
Vernaya	0.002	0.052	-0.021	-0.005	-0.063	0.004	-0.002	-0.007	-0.009
Zelotomys	0.020	0.042	-0.005	-0.023	-0.045	0.001	-0.029	0.001	-0.013
Zyzomys	0.058	0.051	0.001	-0.016	-0.058	0.026	-0.008	-0.010	-0.008
Anthracomys	0.046	0.060	-0.008	0.002	-0.043	0.014	-0.020	-0.016	-0.005
Castillomys	0.041	0.045	-0.002	-0.008	-0.042	0.021	-0.008	-0.026	-0.007
Castromys	0.030	0.048	-0.006	-0.002	-0.024	0.014	-0.017	-0.016	-0.007
Huerzelerimys	0.026	0.051	-0.002	-0.008	-0.038	0.012	-0.017	-0.023	-0.006
Occitanomys	0.046	0.040	-0.002	-0.012	-0.033	0.024	-0.013	-0.029	-0.008
Paraethomys	0.024	0.050	-0.003	-0.012	-0.036	0.015	-0.018	-0.022	-0.004
Progonomys	0.040	0.045	-0.007	-0.007	-0.039	0.020	-0.018	-0.028	-0.005
Rhagapodemus	0.027	0.052	-0.001	-0.006	-0.057	0.010	-0.009	-0.015	-0.011
Stephanomys	0.034	0.045	0.000	-0.017	-0.019	0.023	-0.012	-0.020	0.003

Appendix D.

Genus	A5	B5	C5	D5	A6	B6	C6	D6	A7
Abditomys	0.016	0.004	0.007	-0.014	0.010	-0.007	-0.001	0.016	0.001
Aethomys	0.009	0.008	-0.005	-0.005	0.012	0.001	-0.007	0.000	0.001
Anisomys	-0.002	0.005	-0.004	0.009	0.004	-0.006	0.001	0.007	0.000
Apodemus	0.006	0.004	-0.012	0.002	0.008	0.000	-0.006	-0.008	0.000
Apomys	0.012	0.007	-0.005	-0.016	0.005	-0.003	-0.009	0.002	0.001
Archboldomys	0.001	-0.004	-0.007	0.019	0.009	-0.017	-0.035	0.015	0.007
Arvicanthis	0.015	-0.004	0.004	0.001	0.007	0.002	-0.015	0.004	0.009
Bandicota	0.007	0.001	-0.003	0.008	0.003	-0.007	-0.009	0.000	0.007
Bunomys	0.011	-0.003	0.000	0.000	0.007	-0.005	-0.018	0.005	0.008
Chiropodomys	0.004	0.008	-0.010	-0.004	0.005	0.000	-0.003	0.001	0.000
Chrotomys	0.012	0.001	-0.001	0.006	0.004	-0.010	-0.013	0.004	0.004
Coccymys	0.000	0.004	-0.004	0.000	0.007	-0.009	-0.012	-0.002	-0.001
Colomys	0.006	-0.001	0.002	0.000	0.010	0.000	-0.008	0.001	0.000
Crateromys	0.004	0.007	-0.023	-0.008	0.008	0.000	-0.008	-0.011	-0.007
Crunomys	0.005	0.007	-0.014	0.006	0.010	-0.009	-0.018	0.004	0.001
Dasymys	0.013	-0.005	-0.005	0.005	0.001	-0.005	-0.005	0.009	0.008
Echiothrix	0.006	0.002	0.002	0.001	0.006	0.001	-0.007	0.004	0.003
Eropeplus	0.020	0.012	-0.008	-0.003	0.005	-0.002	-0.015	0.001	0.001
Golunda	-0.003	-0.003	-0.006	-0.004	-0.019	-0.016	0.009	-0.003	-0.002
Grammomys	0.007	0.005	-0.006	0.000	0.004	0.000	-0.010	-0.005	0.001
Hadromys	0.021	-0.003	-0.008	-0.006	0.008	-0.002	-0.013	0.019	0.006
Haeromys	0.008	0.015	-0.008	0.005	0.002	-0.006	-0.006	0.005	0.002
Hapalomys	-0.011	0.002	0.003	0.016	0.008	0.003	-0.002	0.002	0.000
Heimyscus	0.010	0.011	-0.010	0.002	0.016	-0.002	-0.023	0.007	0.001
Hybomys	0.009	0.011	-0.014	-0.004	0.005	0.005	-0.010	-0.007	0.002
Hydromys	0.014	0.002	0.005	-0.014	0.006	-0.009	-0.013	0.015	0.002
Hylomyscus	0.003	0.006	-0.004	-0.016	0.013	-0.005	-0.007	-0.008	-0.007
Hyomys	0.003	0.005	-0.008	-0.002	0.007	-0.008	-0.006	0.006	-0.001
Kadarsanomys	0.020	0.009	0.008	0.000	0.009	0.010	-0.009	0.010	0.007
Leggadina	0.009	0.012	-0.001	-0.011	0.002	-0.001	0.001	-0.004	-0.001
Lemniscomys	0.009	0.003	-0.003	-0.002	0.009	0.000	-0.016	0.004	0.004
Lenomys	0.001	0.009	-0.013	-0.004	0.006	0.001	-0.003	-0.006	-0.003
Lenothrix	0.008	0.007	-0.009	0.013	0.009	-0.001	-0.002	-0.004	0.000
Leopoldomys	0.007	0.010	-0.011	-0.015	0.005	-0.004	-0.004	0.003	-0.002
Leporillus	0.002	0.000	0.004	0.017	0.005	-0.005	-0.014	-0.001	0.009
Leptomys	0.012	0.005	0.014	-0.001	0.006	-0.012	-0.015	0.006	0.004
Lorentzimys	0.006	0.014	-0.014	-0.005	-0.001	-0.002	-0.013	-0.009	-0.002
Malacomys	0.011	0.001	-0.006	-0.005	0.007	0.000	-0.012	-0.004	0.001
Mallomys	0.004	0.016	-0.028	-0.005	0.003	0.007	-0.011	0.001	-0.006
Margaretamys	0.014	0.005	-0.001	-0.005	0.005	-0.002	-0.015	0.001	0.001
Mastacomys	-0.003	-0.006	0.010	0.004	0.003	-0.014	-0.016	0.011	-0.001
Mastomys	0.015	0.005	-0.007	-0.003	0.010	-0.005	-0.021	0.005	0.005
Maxomys	0.006	0.010	-0.008	0.000	0.008	-0.003	-0.010	-0.010	-0.003

Genus	A5	B5	C5	D5	A6	B6	C6	D6	A7
Melasmothrix	0.012	0.017	-0.019	-0.012	0.003	-0.003	-0.018	-0.010	-0.003
Melomys	-0.004	0.006	-0.004	-0.005	0.007	-0.003	-0.002	-0.003	-0.005
Micromys	0.002	0.004	-0.016	-0.023	0.000	0.009	-0.010	-0.006	-0.005
Millardia	0.020	0.009	-0.016	0.000	0.002	0.003	-0.020	-0.009	0.011
Mus	0.016	0.012	-0.002	-0.014	0.005	0.005	-0.007	-0.009	0.001
Nivivemter	0.011	0.004	-0.007	-0.007	0.007	-0.004	-0.015	0.003	0.002
Notomys	0.011	0.008	-0.004	-0.005	0.007	-0.005	-0.008	0.001	0.001
Oenomys	0.009	0.014	-0.010	-0.004	0.015	0.000	-0.012	-0.008	-0.003
Papagonomys	0.017	0.009	-0.008	0.006	-0.001	0.003	-0.016	-0.009	0.009
Parahydromys	0.009	-0.001	0.003	-0.013	0.002	0.005	0.018	0.011	0.000
Paulamys	0.031	-0.008	0.000	-0.005	0.002	0.003	-0.018	-0.001	0.011
Pelomys	0.004	-0.003	-0.005	0.010	0.015	-0.005	-0.014	0.007	0.001
Phloeomys	-0.008	0.007	-0.010	-0.008	0.004	-0.004	0.000	-0.004	-0.002
Pitecheir	0.019	0.015	-0.021	-0.004	0.010	-0.006	0.006	-0.018	0.001
Pogonomys	0.013	0.007	-0.002	0.011	0.008	-0.009	-0.002	-0.005	0.011
Praomys	0.011	0.002	-0.011	-0.014	0.012	-0.002	-0.014	-0.004	0.001
Pseudohydromys	-0.008	0.007	0.006	0.010	0.021	-0.007	-0.014	-0.018	-0.007
Pseudomys	0.008	0.009	-0.009	0.008	-0.005	-0.001	-0.011	-0.010	0.000
Rattus	0.015	0.002	-0.010	-0.005	0.008	-0.003	-0.016	0.000	0.004
Rhabdomys	0.005	0.009	-0.005	-0.011	0.008	0.003	-0.007	-0.011	-0.002
Rhynchomys	0.011	0.009	0.007	-0.025	-0.004	-0.004	-0.009	-0.001	0.003
Solomys	0.009	-0.002	0.000	-0.009	0.005	0.002	-0.003	0.001	-0.003
Sommeromys	0.010	-0.003	0.001	-0.006	0.001	-0.004	-0.018	-0.002	0.002
Spelaeomys	0.000	0.001	-0.014	0.004	-0.008	0.002	0.000	0.005	-0.002
Stochomys	0.006	0.004	-0.004	0.002	-0.002	-0.003	-0.007	0.001	0.006
Sundamys	0.016	0.003	-0.008	-0.003	0.006	-0.003	-0.009	0.000	0.003
Tateomys	0.019	-0.005	0.007	0.005	0.013	-0.003	-0.022	-0.008	0.007
Thallomys	0.007	0.009	-0.006	-0.003	0.009	-0.001	-0.012	-0.010	0.001
Thammomys	0.002	0.007	-0.009	0.003	0.010	-0.003	-0.016	0.001	-0.001
Tokudaia	0.001	0.003	-0.006	0.015	-0.012	-0.003	-0.008	0.000	0.007
Uromys	0.009	0.002	-0.008	-0.011	0.004	-0.004	-0.011	-0.002	-0.001
Vandeluria	0.001	0.006	-0.028	-0.016	0.005	0.002	0.000	-0.015	-0.005
Vernaya	0.018	-0.003	-0.021	0.007	-0.004	0.000	-0.001	0.002	0.008
Zelotomys	0.000	0.000	0.017	-0.008	0.005	-0.019	-0.014	0.020	0.003
Zyzomys	0.002	0.008	-0.002	-0.012	0.010	0.000	-0.004	-0.012	-0.002
Anthracomys	0.021	-0.003	-0.008	0.007	0.005	-0.003	-0.009	-0.013	0.010
Castillomys	0.009	0.010	-0.012	-0.003	0.002	0.005	-0.005	-0.012	0.005
Castromys	0.016	0.000	-0.006	-0.003	0.003	0.002	-0.010	-0.006	0.003
Huerzelerimys	0.017	0.006	-0.007	0.003	0.002	0.003	-0.011	-0.005	0.006
Occitanomys	0.012	0.009	-0.009	0.000	0.004	0.005	-0.004	-0.011	0.006
Paraethomys	0.018	0.006	-0.010	0.002	0.006	0.003	-0.012	-0.002	0.004
Progonomys	0.013	0.004	-0.011	-0.003	0.006	0.005	-0.011	-0.008	0.003
Rhagapodemus	0.005	0.008	-0.010	-0.007	0.001	0.001	-0.005	-0.004	-0.001
Stephanomys	0.023	0.010	-0.007	0.004	0.006	0.003	-0.005	-0.011	0.011

Appendix D.

Genus	B7	C7	D7	A8	B8	C8	D8	A9	B9
Abditomys	-0.001	-0.006	-0.005	0.011	-0.002	-0.003	-0.012	-0.004	0.006
Aethomys	0.004	0.007	-0.005	0.001	0.002	0.004	-0.012	0.000	-0.001
Anisomys	0.003	0.004	0.001	0.004	-0.003	-0.001	0.000	0.002	0.001
Apodemus	0.007	0.000	-0.005	0.002	-0.003	0.012	0.001	-0.002	0.002
Apomys	0.006	-0.001	-0.001	0.002	-0.002	0.006	-0.007	-0.001	0.003
Archboldomys	0.005	-0.005	-0.004	0.007	-0.002	0.010	-0.011	0.003	0.005
Arvicanthis	0.001	-0.006	-0.005	-0.002	0.005	0.009	-0.016	0.000	0.002
Bandicota	0.002	-0.001	-0.001	-0.002	0.002	-0.002	-0.005	0.001	0.000
Bunomys	0.001	-0.006	0.001	-0.001	0.002	0.007	-0.016	0.001	0.002
Chiropodomys	0.009	0.000	0.001	0.001	-0.002	0.004	-0.003	-0.001	0.004
Chrotomys	0.004	-0.007	-0.011	0.002	-0.002	0.002	-0.004	0.000	0.003
Coccymys	0.006	-0.006	-0.003	0.002	-0.003	0.005	-0.007	-0.003	0.003
Colomys	0.002	-0.004	-0.001	0.002	0.000	0.007	-0.014	0.000	0.002
Crateromys	0.009	0.002	-0.005	0.003	-0.001	0.004	0.006	-0.003	-0.001
Crunomys	0.007	0.004	-0.014	0.004	0.000	0.008	-0.008	-0.005	0.003
Dasymys	0.003	-0.006	-0.005	0.001	0.002	-0.005	-0.006	0.000	0.002
Echiothrix	0.002	-0.008	0.000	0.005	0.002	0.006	-0.002	-0.001	0.002
Eropeplus	0.008	0.015	-0.011	0.001	0.001	0.009	-0.012	0.000	0.000
Golunda	0.000	0.003	0.007	-0.009	-0.015	-0.007	0.011	0.003	-0.004
Grammomys	0.004	0.003	-0.004	0.000	-0.001	0.011	-0.001	-0.002	0.000
Hadromys	0.004	-0.003	-0.011	0.007	0.004	0.001	-0.010	-0.004	0.001
Haeromys	0.010	0.007	-0.009	-0.001	-0.001	0.001	-0.012	0.000	0.004
Hapalomys	0.002	-0.004	0.009	0.007	0.003	0.004	-0.003	0.003	0.002
Heimyscus	0.009	0.013	-0.003	0.006	0.002	0.017	-0.019	-0.004	0.004
Hybomys	0.006	0.015	-0.001	0.001	-0.001	0.016	0.003	-0.001	-0.002
Hydromys	0.003	-0.010	-0.007	0.006	-0.002	0.005	-0.013	-0.003	0.003
Hylomyscus	0.006	0.002	-0.005	0.005	-0.004	0.002	-0.003	-0.004	0.000
Hyomys	0.004	0.008	-0.005	0.004	-0.002	-0.001	-0.009	0.000	0.002
Kadarsanomys	0.001	0.004	-0.012	0.001	0.004	0.016	-0.022	-0.001	0.000
Leggadina	0.003	0.015	-0.001	0.000	-0.002	0.001	0.000	0.000	-0.003
Lemniscomys	0.003	0.003	-0.002	0.001	0.004	0.006	-0.013	0.000	0.000
Lenomys	0.007	0.003	-0.001	0.003	-0.001	0.006	0.006	0.002	0.003
Lenothrix	0.010	0.001	-0.007	0.003	0.001	0.010	-0.005	-0.004	0.002
Leopoldomys	0.010	0.006	-0.002	0.000	0.000	-0.003	-0.010	-0.003	0.003
Leporillus	0.003	-0.008	0.001	0.000	0.007	-0.003	-0.004	0.003	0.003
Leptomys	0.005	-0.010	0.002	0.000	-0.003	0.005	-0.012	0.002	0.005
Lorentzimys	0.008	0.012	-0.001	-0.002	0.001	0.005	0.005	-0.002	0.000
Malacomys	0.005	0.001	-0.005	0.000	0.001	0.008	-0.008	-0.001	0.003
Mallomys	0.007	0.033	-0.002	0.001	0.002	0.009	0.000	-0.002	-0.004
Margaretamys	0.005	-0.002	-0.008	0.001	-0.001	0.012	-0.009	-0.002	0.004
Mastacomys	-0.001	-0.012	-0.004	0.005	0.003	-0.004	-0.008	-0.005	0.002
Mastomys	0.008	0.006	0.000	0.001	0.000	0.008	-0.020	0.001	0.005
Maxomys	0.006	0.012	-0.009	0.001	-0.001	0.007	-0.003	-0.003	0.000

Genus	B7	C7	D7	A8	B8	C8	D8	A9	B9
Melasmothrix	0.006	0.036	0.003	0.004	0.006	0.007	-0.010	-0.002	-0.011
Melomys	0.004	0.002	0.002	0.001	-0.001	0.003	-0.007	-0.003	0.001
Micromys	0.002	0.016	0.007	0.001	0.003	0.012	0.008	-0.002	-0.003
Millardia	0.008	0.011	-0.004	-0.006	0.001	0.016	-0.007	0.003	0.000
Mus	0.003	0.011	0.001	0.001	-0.001	0.011	-0.005	0.002	0.000
Nivivemter	0.006	-0.003	-0.004	0.001	-0.001	0.007	-0.011	0.000	0.003
Notomys	0.006	0.003	-0.005	0.001	0.001	0.001	-0.008	-0.002	0.001
Oenomys	0.005	0.015	-0.013	0.002	0.003	0.006	-0.007	-0.007	-0.004
Papagonomys	0.006	0.007	-0.009	-0.001	0.000	0.023	0.006	0.001	-0.003
Parahydromys	-0.002	-0.001	0.011	0.003	0.000	-0.008	-0.005	0.002	-0.001
Paulamys	-0.004	-0.003	-0.007	0.000	0.000	0.015	-0.003	0.003	-0.001
Pelomys	0.003	-0.001	-0.010	0.004	0.003	0.001	-0.012	-0.004	0.001
Phloeomys	0.004	0.006	0.010	0.001	-0.001	-0.004	0.001	0.002	0.000
Pitecheir	0.014	0.017	-0.012	-0.002	-0.008	0.003	-0.010	-0.001	0.002
Pogonomys	0.003	0.002	-0.006	0.001	-0.002	-0.002	-0.004	0.000	0.000
Praomys	0.007	0.001	0.000	0.002	0.000	0.009	-0.010	0.000	0.002
Pseudohydromys	0.006	-0.003	-0.006	0.007	0.000	0.010	0.000	-0.004	0.000
Pseudomys	0.004	0.008	-0.012	-0.005	0.003	0.005	0.007	-0.006	-0.002
Rattus	0.004	0.003	-0.008	0.000	0.001	0.006	-0.012	-0.001	0.001
Rhabdomys	0.001	0.012	-0.001	0.001	0.002	0.001	0.000	-0.001	-0.003
Rhynchomys	0.002	0.005	0.002	0.000	0.002	0.001	0.003	0.002	-0.001
Solomys	0.002	-0.005	-0.008	0.001	-0.001	0.008	-0.006	-0.005	0.001
Sommeromys	-0.002	-0.002	0.000	-0.002	-0.001	0.009	-0.002	0.001	0.002
Spelaeomys	0.009	-0.004	-0.003	-0.001	0.001	0.002	0.007	-0.003	0.004
Stochomys	0.004	0.001	-0.001	-0.004	0.000	0.002	-0.005	0.003	0.003
Sundamys	0.003	0.006	-0.008	0.001	-0.001	0.008	-0.009	-0.001	-0.001
Tateomys	-0.002	-0.010	-0.023	-0.001	0.003	0.015	-0.011	-0.007	0.002
Thallomys	0.005	0.007	-0.003	0.001	0.001	0.011	0.002	-0.001	-0.003
Thammomys	0.010	-0.002	-0.006	0.004	0.002	0.008	-0.005	-0.004	0.002
Tokudaia	-0.003	0.012	-0.003	-0.006	0.004	0.004	0.007	0.001	-0.002
Uromys	0.003	0.002	-0.004	0.002	-0.001	0.004	0.000	-0.003	0.001
Vandeluria	0.006	0.010	0.002	0.001	-0.002	0.008	0.002	-0.002	0.000
Vernaya	0.009	-0.009	-0.014	-0.004	-0.001	0.005	0.005	-0.005	0.006
Zelotomys	-0.005	-0.007	0.015	0.008	-0.002	-0.008	-0.015	0.005	0.003
Zyzomys	0.005	0.007	0.002	-0.001	-0.003	0.010	-0.006	-0.001	-0.001
Anthracomys	0.000	0.006	-0.008	-0.004	0.001	0.003	0.001	0.001	-0.002
Castillomys	0.004	0.010	0.004	0.000	0.001	0.011	0.004	0.002	-0.001
Castromys	0.002	0.002	-0.008	-0.002	-0.001	0.011	-0.002	-0.002	0.000
Huerzelerimys	0.003	0.007	-0.008	-0.002	0.001	0.013	-0.003	0.000	0.000
Occitanomys	0.004	0.008	0.000	-0.002	0.000	0.011	0.000	0.002	0.000
Paraethomys	0.004	0.010	-0.011	0.000	0.000	0.015	-0.006	-0.002	-0.001
Progonomys	0.004	0.007	-0.001	-0.001	0.001	0.013	-0.004	0.000	-0.001
Rhagapodemus	0.005	0.006	0.000	0.000	-0.002	0.010	0.004	-0.001	0.000
Stephanomys	0.003	0.011	-0.007	-0.003	-0.001	0.010	-0.005	0.003	0.000

Appendix D.

Genus	C9	D9
Abditomys	-0.005	-0.008
Aethomys	-0.002	0.002
Anisomys	0.000	-0.001
Apodemus	0.002	-0.004
Apomys	0.003	-0.003
Archboldomys	-0.002	0.000
Arvicanthis	0.001	-0.004
Bandicota	0.002	-0.004
Bunomys	0.002	-0.003
Chiropodomys	0.005	-0.004
Chrotomys	0.005	0.003
Coccymys	0.005	0.002
Colomys	0.000	0.001
Crateromys	0.010	0.002
Crunomys	0.001	0.006
Dasymys	0.005	-0.003
Echiothrix	0.006	-0.006
Eropeplus	-0.007	0.007
Golunda	0.003	0.000
Grammomys	0.001	-0.002
Hadromys	0.008	-0.004
Haeromys	0.001	0.005
Hapalomys	0.001	-0.006
Heimyscus	-0.009	-0.001
Hybomys	-0.003	-0.004
Hydromys	0.001	0.002
Hylomyscus	0.003	0.008
Hyomys	-0.004	0.009
Kadarsanomys	-0.002	0.001
Leggadina	-0.004	0.002
Lemniscomys	0.000	0.000
Lenomys	0.005	0.005
Lenothrix	0.004	-0.008
Leopoldomys	0.005	0.003
Leporillus	0.004	-0.006
Leptomys	0.005	-0.005
Lorentzimys	-0.002	0.001
Malacomys	-0.003	0.001
Mallomys	-0.013	0.002
Margaretamys	0.000	0.002
Mastacomys	0.008	0.001
Mastomys	-0.005	-0.001
Maxomys	-0.007	0.007

Genus	C9	D9
Melasmothrix	-0.016	-0.003
Melomys	0.002	-0.001
Micromys	-0.007	-0.003
Millardia	-0.004	-0.003
Mus	-0.007	-0.003
Nivivemter	0.003	0.003
Notomys	0.001	0.000
Oenomys	0.000	0.003
Papagonomys	0.004	-0.002
Parahydromys	-0.005	-0.004
Paulamys	-0.004	-0.004
Pelomys	0.001	0.004
Phloeomys	-0.001	-0.003
Pitecheir	-0.006	0.004
Pogonomys	0.001	-0.010
Praomys	0.002	-0.001
Pseudohydromys	0.002	0.001
Pseudomys	0.000	0.001
Rattus	0.000	0.002
Rhabdomys	-0.004	0.000
Rhynchomys	0.003	0.002
Solomys	0.003	0.003
Sommeromys	-0.003	-0.001
Spelaeomys	0.006	0.000
Stochomys	-0.001	0.001
Sundamys	0.000	0.000
Tateomys	0.001	0.006
Thallomys	0.003	-0.003
Thammomys	0.010	-0.001
Tokudaia	-0.007	0.000
Uromys	0.001	0.003
Vandeluria	0.002	0.003
Vernaya	0.021	-0.012
Zelotomys	-0.006	-0.004
Zyzomys	0.000	0.001
Anthracomys	-0.007	-0.006
Castillomys	-0.002	-0.010
Castromys	-0.002	-0.001
Huerzelerimys	-0.004	-0.003
Occitanomys	-0.002	-0.007
Paraethomys	-0.005	-0.001
Progonomys	-0.002	-0.004
Rhagapodemus	0.001	0.000
Stephanomys	-0.008	-0.007
